# DEFEAT: Android device behavior-based datasets for multi-stage APT

**DOI:** 10.1016/j.dib.2026.112539

**Published:** 2026-02-10

**Authors:** Thulfiqar Jabar, Amjed Ahmed Al-Kadhimi, Manmeet Mahinderjit Singh

**Affiliations:** a School of Computer Sciences, Universiti Sains Malaysia, Georgetown 11800, Penang, Malaysia; b College of Medicine, Al-Muthanna University, Al Samawah 66001, Iraq; c Department of Computer Engineering, College of Engineering, University of Basrah, Basrah 61004, Iraq

**Keywords:** Device behavior analysis, Resource usage features, App-based features, Dataset generation, Advanced persistent threat (APT), Android security. mobile cyberattacks

## Abstract

Android devices play a central role in both personal and organizational operations, which has made them a primary target for Advanced Persistent Threats (APTs). Unlike traditional attacks, APT attacks are implemented through multiple covert stages, allowing attackers to remain active on a device while avoiding detection models. Existing studies depend on data that captures only a single stage of an attack or focuses mainly on static features. Consequently, detection models trained on such datasets may fail to detect multi-stage APT attacks in real-world environments. In order to address this gap, this paper introduces DEFEAT, a benchmarking dataset built specifically for detecting APT attacks on Android devices. DEFEAT follows the MITRE ATT&CK framework to more accurately reflect multi-stage APT attacks in real-world environments. The dataset generation process includes three main phases: gathering normal activity, simulating multi-stage APT attacks, and preparing the data. The datasets were collected from a real Android smartphone and are provided in two parts: a resource-usage dataset that tracks CPU, RAM, battery, and network activity; and an app-based dataset that logs permissions, sensors, and services used by apps. The dataset captures the active phase of APT attacks, focusing on observable malicious behavior rather than long-term dormant activity. The requirements of a well-structured dataset have been met in the proposed datasets to ensure they are suitable for use by other researchers. Feature contributions have also been examined using SHAP (SHapley Additive exPlanations) to better understand their role in detecting APTs. In addition, statistical t-test analysis is applied to the resource-usage datasets to verify that the collected behavioral features vary significantly across malware families and attack stages, supporting their suitability for behavior-based APT detection. By offering a realistic and publicly accessible representation of multi-stage APTs, DEFEAT addresses an important gap in current Android security research and supports the development of more effective behavioral detection models. The datasets are publicly available and can be reused by other researchers for the tuning, evaluation, and comparison of detection models for multi-stage APT activities on Android devices.

Specifications Table

**Table:** 

Subject	Computer Sciences
Specific subject area	APT Android dataset that includes two parts: resource usage (CPU, RAM, battery, network traffic) and app-based features (permissions, sensors, services).
Type of data	Raw: csv files. Data format: resource usage features (8 features per frame), app-based features (108 features per frame).
Data collection	Datasets were collected using the DEFENSE Android data-collection application deployed on a real smartphone (Android version 6). It runs the DEFENSE collector that collects resource usage (CPU, RAM, battery activity, and network traffic) and app-based features (permissions, sensors, and services) and sends them to the server every 3 seconds, then exports them to CSV. A total of 40 normal apps were used as a baseline, and 36 malwares were used to simulate the APT stages after VirusTotal validation, with device reset between runs. Each app was executed for 10 minutes, the records were stage-labelled, and the data were stored on the server.
Data source location	School of Computer Sciences, Universiti Sains Malaysia, 11800 USM Penang, Malaysia.
Data accessibility	Repository name: [Mendeley Repository] [1]. Data identification number: 10.0.68.224/bdtn9vj7d7.3 Direct URL to data: https://doi.org/10.17632/bdtn9vj7d7.3
Related research article	None

## 1 Value of the Data

• Comprehensive behavioral coverage: The DEFEAT datasets bring together both static and dynamic features, from how apps behave to how the device uses its resources and network. By combining these features, they offer a clear, detailed view of how APT attacks influence an Android device at each stage of the attack.
• Active-phase APT behavior: The DEFEAT datasets are designed to capture the active phase of APT attacks, during which malicious actions such as privilege misuse, credential access, and data exfiltration occur. Rather than modelling long-term dormancy or dwell time, the dataset emphasizes observable behavioral deviations that are most relevant for practical detection systems.
• Realistic data collection: The DEFEAT datasets were captured from a physical Android device, rather than synthesized by emulator tools. Therefore, the datasets are far more realistic and reliable because they align perfectly with the behavior of an actual end user.
• Explicit multi-stage APT simulation: The DEFEAT datasets carefully simulate how APTs progress step by step, using the MITRE ATT&CK framework. Abnormal activities during Initial Compromise, Credential Access, and Exfiltration stages are simulated in the resource-usage dataset; while the app-based dataset simulates the attack path: Initial Compromise, Privilege Escalation, and Exfiltration. This structural design gives a solid basis for analyzing multi-stage attack behaviors in detail.
• Broad applicability: These datasets are valuable resources for cybersecurity researchers, data scientists and system developers seeking to evaluate and benchmark APT detection systems on Android devices.

## 2 Background

With the widespread adoption of smartphones, Android has become the most widely used Operating System globally. Nevertheless, due to its open-source nature and broad application ecosystem have made it a prime target for cyberattacks. Among these attacks, Advanced Persistent Threats (APTs) are the most sophisticated and harmful attacks, in which attackers design covert application malware implemented in multiple stages to evade detection. While existing datasets provide a high-level view of multi-stage APT attacks, their dependency on static features overlooks the dynamic behavioral patterns showcased by APT attacks. Consequently, detection systems trained on such datasets may fail to detect multi-stage APT attacks in real-world environments. Therefore, there is a clear need for reference-labelled datasets that capture multi-stage APT activities using representative features for evaluation and comparison purposes. This paper presents DEFEAT, the first labelled multi-stage APT dataset, collected from physical Android devices, that integrates both static and dynamic features. The DEFEAT datasets consist of two complementary parts: a resource-usage dataset with CPU, RAM, battery, and traffic features, and an app-based dataset with permissions, sensors, and services. The DEFEAT dataset focuses on capturing the active phase of APT attacks, during which malicious actions such as privilege misuse, credential access, and data exfiltration occur. While real-world APT campaigns may include extended dormant periods, detection systems typically rely on observable behavioral deviations that arise when malware becomes operational. Accordingly, DEFEAT is designed to benchmark detection performance during these active phases, where measurable device-level and application-level behaviors emerge. These datasets have been rigorously validated and evaluated to ensure their suitability for use by other researchers in tuning, testing, and comparing detection models.

## 3 Data Description

DEFEAT datasets is available at [1]. The DEFEAT datasets were generated from a physical Android device which consists of two complementary components: a Resource-usage dataset and App-based dataset. Each dataset consists of three CSV files, each corresponding to a specific stage of attack. Both of these datasets are generated concurrently and follow the common APT attack paths, starting with the Initial Compromise stage and ending with the Exfiltration stage [2]. The detailed characteristics of these datasets are: a. Resource-usage dataset: This dataset consists of 8 features which are divided into five categories including: App-Info, Battery-Info, CPU-Info, RAM-Info, and Traffic. This dataset consists of three csv files as follows:

• Resource usage - Initial Compromise.csv: This dataset represents APT activities during Initial Compromise stage. It contains 12,741 frames, of which 6,209 correspond to abnormal data and 6,532 to normal data.
• Resource usage - Credential Access.csv: This dataset represents APT activities during Credential Access stage. It contains 12,761 frames, of which 6,229 correspond to abnormal data and 6,532 to normal data.
• Resource usage – Exfiltration.csv: This dataset represents APT activities during Exfiltration stage. It contains 12,833 frames, of which 6,301 corresponds to abnormal data and 6,532 to normal data.

Table 1 provides a concise overview of the DEFEAT datasets, including the number of files per dataset, sampling unit, feature groups with their dimensionality, and associated data types for both resource-usage and app-based components.

**Table 1:** Summary of DEFEAT dataset components, feature groups, dimensionality, and data types.

Dataset	No, of files	Sampling unit	Feature Group	Description	No. of Features	Data Type
Resource-usage dataset	3 CSV files	1 frame / 3 seconds	App-Info	Application data size (MB)	1	Numeric (float)
			Battery-Info	Battery voltage and temperature (%)	2	Numeric (float)
			CPU-Info	Device-level CPU usage (%)	1	Numeric (float)
			RAM-Info	Device-level RAM usage (MB)	1	Numeric (float)
			Traffic	RX, TX, and total traffic (MB)	3	Numeric (float)
			Class label	0 = normal, 1 = abnormal	-	Integer (0/1)
Total		8				

App-based dataset	3 CSV files	1 frame / 3 seconds	Sensors	Camera, GPS, Microphone, Wi-Fi, Bluetooth	5	Binary (0/1)
			Services	SMS, Phone, Contacts, Storage, Calendar	5	Binary (0/1)
			Permissions	Encoded as permission-usage indicators per frame; names include tier weights (e.g.,0.25/0.5/0.75/1.0) indicating permission category/level	98	Binary (0/1)
			Class label	0 = normal, 1 = abnormal	-	Integer (0/1)
Total		118				

**Fig. 1:**
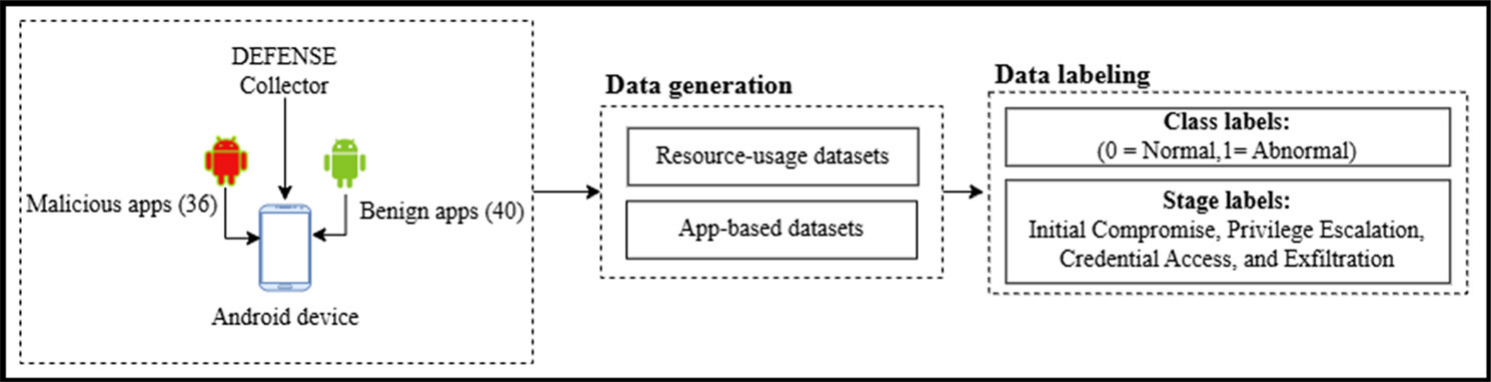
Data generation and labelling pipelines.

Fig. 1 illustrates the DEFEAT data generation and labelling pipeline, starting from benign and malicious Android applications, followed by behavioral data collection using the DEFENSE collector. The collected data are then organized into two components: resource-usage datasets and app-based datasets. Finally, deterministic labelling is applied, including binary class labels (normal vs. abnormal) and stage labels corresponding to the simulated APT stage.

A detailed description of these features is provided in Table 2. b. App-based dataset: This dataset consists of 108 features and contains permissions, sensors and services features. This dataset consists of three csv files as follows:

• Stg1.initial.csv: This dataset represents APT activities during Initial Compromise stage. It contains 12,741 frames, of which 6,209 correspond to abnormal data and 6,532 to normal data.
• Stg2.privilege.csv: This dataset represents APT activities during privilege escalation stage. It contains 12,761 frames, of which 6,229 correspond to abnormal data and 6,532 to normal data.
• Stg3.exfiltration.csv: This dataset represents APT activities during Exfiltration stage. It contains 12,833 frames, of which 6,301 corresponds to abnormal data and 6,532 to normal data.

**Table 2:** Resource usage features with their description.

Feature category	Feature name	Description
App-Info	App-data-size	The data size of normal and abnormal apps, measured in megabytes.
Battery-Info	B-temperature	The temperature of the device’s battery, often monitored to prevent overheating.
	B-voltage	The voltage level of the device's battery.
CPU-Info	CPU-Usage	The amount of processing power is currently being used by the device’s central processing unit (CPU).
RAM-Info	RAM-Usage	The amount of Random Access Memory (RAM) is being used by the device to run apps and processes.
Traffic	Received Data (RX)	The amount of data that has been downloaded from the internet to the device, measured in megabytes.
	Transferred Data (TX)	The amount of data that has been uploaded from the device to the internet, measured in megabytes.
	Traffic	The total amount of data traffic, both sent and received, on the device, measured in megabytes.

For both datasets, normal instances (Class 0) are recorded by observing the behavior of 40 legitimate apps for one day. While attack instances (Class 1) are recorded by observing the behavior of 36 malicious apps for three days, one day for each attack stage. In addition, each normal/malicious app was executed in the device for 10 minutes, and the data is collected using specific app collector called DEFENSE, which transmits the data every 3 seconds to a remote server.

Table 3 provides an explanation of a part of these features and their description (full features description in Table A1, Table A2) (Table 4, Table 5, Table 6).

**Table 3:** App permissions, sensors, and services features with their description.

Features	Description
Camera	Ambient sensor, Access camera and capture image and video
GPS	Positioning sensor, Location tracking and transmission of information
Microphone	Ambient sensor, Access microphone record audio
WIFI	Positioning sensor, Location tracking and transmission of information
Bluetooth	Positioning sensor, Location tracking and transmission of information
SMS	Telephony services/ Access and send messages
Phone	Telecommunication, Access to telephony functions such as retrieving contact numbers, managing call states, and monitoring device telephony status
Contacts	Telecommunication/ Access contacts and profiles
Storage	Utilities/ Access to external storage
Calendar	Utilities/ Access and modify user calendar data
Normal Permissions	
access_location_extra_commands	Grants access to advanced location provider commands
access_network_state	Grants applications the ability to retrieve information regarding active network connections
access_wifi_state	Allows retrieval of Wi-Fi network details, such as SSID, signal strength, and connectivity status
bluetooth_admin	Grants apps the ability to discover nearby Bluetooth devices and initiate pairing
Dangerous Permissions	
access_background_location	Grants apps the ability to access location in the background
access_coarse_location	Grants apps the ability to access approximate location
access_fine_location	Grants apps the ability to access precise location
access_media_location	Grants applications access to stored geographic location data shared by the user or persisted across services
activity_recognition	Grants apps the ability to recognize physical activity
Signature Permissions	
bind_accessibility_service	Required by an Accessibility Service to ensure binding is restricted to the system, protecting against unauthorized access or misuse
broadcast_sms	Grants app the ability to broadcast a notification upon receipt of an SMS message
capture_audio_output	Grants an app the ability to capture or record audio being played by the device
change_component_enabled_state	Grants an app the ability to change whether an app component is enabled or not
delete_packages	Grants an app the ability to delete package
Privileged Permissions	
battery_stats	Grants an app the ability to collect battery statistics
call_privileged	Grants an app the ability to initiate phone calls, including emergency numbers without user interaction or confirmation via the Dialer interface
change_configuration	Grants an app the ability to alter system configuration settings
get_accounts_privileged	Grants apps access to the list of user accounts registered on the device via the Accounts Service
package_usage_stats	Grants an app the ability to collect component usage statistics

**Table 4:** Resource usage features extraction.

App name	Data Size	Battery Temperature	Battery Voltage	CPU Usage	RAM Usage	RX	TX	Traffic
Youla	0.176	31.4	3.747	0.11	0.59	67.17	121	188
Gumtree	0.164	30.1	3.622	0.09	0.63	70.51	141	211
memory booster	0.112	33	4.029	0.12	0.58	12.49	67.27	79.76
PhotoWonder	0.1	31.5	3.635	0.11	0.58	176	137	313
Dendroid	0.02	30.4	4.18	0.13	0.68	2.43	17.55	19.98
Setel	18.86	31.9	3.677	0.34	0.75	1340	386	1720
GoodFM	60.95	33.9	3.996	0.31	0.84	668	58.14	727
Messenger	114	32.2	3.78	0.14	0.78	718	113	831
Gumtree	0.164	30.1	3.431	0.11	0.64	70.52	141	211
WEBTOON	31.35	31.4	3.575	0.38	0.81	703	102	805
DramaBox	29.24	32.7	3.634	0.15	0.86	815	149	940
Nobetci eczane	0.068	31.9	4.073	0.19	0.65	14.25	21.87	36.12
xRecorder	9.36	31	4.052	0.24	0.77	2280	472	2740
nobetci eczane	0.068	32	4.085	0.14	0.64	14.35	23.58	37.93
Elmo loves ABCs	0.156	32.7	3.744	0.27	0.78	990	213	1200
MetaMask	0.088	31.6	3.832	0.05	0.61	56.58	96.03	153
KenanganCoffee	4.45	31	4.088	0.14	0.82	858	175	1010
Youla	0.176	31.2	3.684	0.13	0.59	67.3	124	192
Egypt 3D	0.024	32.3	3.814	0.09	0.57	103	93.35	197
Al Jazeera	6.05	31.3	3.847	0.15	0.76	2340	506	2830
GoogleUpdater	0.092	29.8	3.97	0.15	0.68	18.78	58.59	77.37
DramaBox	29.77	32.6	3.615	0.22	0.88	821	151	950
Chrome	12.54	30.5	3.66	0.11	0.78	2800	540	3330
dragon fighter 3d	3.2	31.5	3.701	0.15	0.57	128	116	243
Chrome	12.55	30.6	3.675	0.09	0.8	2800	540	3330
Chrome	17.18	31.5	3.674	0.21	0.82	2810	548	3340
AlfredCamera	11.31	33.3	3.789	0.29	0.83	990	206	1190
Egypt 3D	0.028	32.3	3.772	0.19	0.58	104	96.52	200
Moomoo	89.23	31.2	3.711	0.21	0.75	2800	531	3320

**Table 5:** Apps sensors and services features extraction.

App name	camera	contacts	GPS	SMS	PHONE	CALENDER	WIFI	BLUETOOTH
Dendroid	1	1	1	1	1	0	1	0
Dendroid	1	1	1	1	1	0	1	0
Dendroid	1	1	1	1	1	0	1	0
Dendroid	1	1	1	1	1	0	1	0
Dendroid	1	1	1	1	1	0	1	0
Dendroid	1	1	1	1	1	0	1	0
Dendroid	1	1	1	1	1	0	1	0
Dendroid	1	1	1	1	1	0	1	0
Dendroid	1	1	1	1	1	0	1	0
Dendroid	1	1	1	1	1	0	1	0
Dendroid	1	1	1	1	1	0	1	0
Dendroid	1	1	1	1	1	0	1	0
Dendroid	1	1	1	1	1	0	1	0
Dendroid	1	1	1	1	1	0	1	0
Dendroid	1	1	1	1	1	0	1	0
Dendroid	1	1	1	1	1	0	1	0
Dendroid	1	1	1	1	1	0	1	0
Dendroid	1	1	1	1	1	0	1	0
Dendroid	1	1	1	1	1	0	1	0
Dendroid	1	1	1	1	1	0	1	0

**Table 6:** Apps permissions features extraction.

App name	flashlight 0.25	access_network_state 0.25	access_notification_policy 0.25	access_wifi_state 0.25	badge_count_read 0.25	billing 0.25	bluetooth 0.25	bluetooth_admin 0.25	broadcast_badge 0.25	broadcast_sticky 0.25
Dendroid	0	1	0	1	0	0	0	0	0	0
Dendroid	0	1	0	1	0	0	0	0	0	0
Dendroid	0	1	0	1	0	0	0	0	0	0
Dendroid	0	1	0	1	0	0	0	0	0	0
Dendroid	0	1	0	1	0	0	0	0	0	0
Dendroid	0	1	0	1	0	0	0	0	0	0
Dendroid	0	1	0	1	0	0	0	0	0	0
Dendroid	0	1	0	1	0	0	0	0	0	0
Dendroid	0	1	0	1	0	0	0	0	0	0
Dendroid	0	1	0	1	0	0	0	0	0	0
Dendroid	0	1	0	1	0	0	0	0	0	0
Dendroid	0	1	0	1	0	0	0	0	0	0
Dendroid	0	1	0	1	0	0	0	0	0	0
Dendroid	0	1	0	1	0	0	0	0	0	0
Dendroid	0	1	0	1	0	0	0	0	0	0
Dendroid	0	1	0	1	0	0	0	0	0	0
Dendroid	0	1	0	1	0	0	0	0	0	0
Dendroid	0	1	0	1	0	0	0	0	0	0
Dendroid	0	1	0	1	0	0	0	0	0	0

Fig. 4, Fig. 5, Fig. 6 show the extracted DEFEAT features including (1) resource usage features and (2) app permissions, sensors, and services features.

### 2.1. Datasets comparison

Table 7 provides a qualitative comparison between the proposed DEFEAT datasets and the most relevant APT-related datasets in the literature. The comparison focuses on five dimensions: dataset focus, feature types, MITRE alignment, multi-stage APT coverage, and online availability. A detailed discussion of these aspects is presented below.

**Table 7:** Qualitative comparison between the proposed DEFEAT datasets and the existing APT datasets.

Reference	Focused	Features	MITRE	Multi-stage coverage	Online availability
[5,6]	Network-centric	URL	No	No	No
[3,4]	Network-centric	DNS logs	No	No	No
[9]	App-centric	Permissions, intents and API calls	No	No	No
[2,10]	Device + Network-centric	System logs, network traces	No	No	Yes
[11]	App + Device-centric	Permissions, activities, Intents, services, receivers, system calls	No	No	Yes
[8]	App-centric	Binary vectors of TTP and IoC (MITRE based)	Yes	Yes	No
[7]	App-centric	Permissions, activities, services, receivers, intents	Yes	Yes	Yes
DEFEAT dataset	Device, Network, and App-centric	•Resource usage (CPU, RAM, battery, traffic) •App sensors permissions, services	Yes	Yes	Yes

#### 3.1.1 Dataset focus

Most existing APT datasets generally provide a limited view of APT behavior. Some are network-centric, relying mainly on URL or DNS traffic logs [[3], [4], [5], [6]]. Others capture only application-level behavior, such as permissions and intents [[7], [8], [9]]. A few datasets provide broader coverage by combining device and network logs [2,10], or by integrating device-level and application-level activities [11].

In contrast, the DEFEAT datasets capture the following features: resource consumption (CPU, RAM, battery), Network-level: RX, TX, and total traffic, App-level: permissions, services, and sensors. By capturing activity across different parts of the device, DEFEAT more accurately reflects how real APTs operate, enabling the modelling of complex behaviors that emerge during an attack.

#### 3.1.2 Feature types

Most existing datasets rely on either static or dynamic features, but rarely both. Static features such as permissions, intents, and API calls, are common in app-centric datasets [[7], [8], [9]]. Dynamic features such as system logs, DNS logs, and traffic traces, are widely used in network- or device-centric datasets [[2], [3], [4], [5], [6],^10^]. However, static-only datasets often fail to capture runtime malicious behaviors, while dynamic-only datasets may overlook an app’s inherent risk. Only one study incorporates both static and dynamic features, including permissions, activities, intents, services, receivers, and system calls [11]. Nevertheless, this dataset still lacks comprehensive coverage of device behavior, network traffic, and app-level activities, which may limit its ability to reliably distinguish between benign and APT behaviors.

The DEFEAT datasets integrate both static and dynamic features, including Resource-usage features (CPU, RAM, battery voltage/temperature, traffic), and Application-behavior features (permissions, sensors, services). By combining these complementary feature groups, DEFEAT provides behavioral indicators capable of distinguishing benign processes from multi-stage APT activities.

#### 3.1.3 MITRE alignment

Most existing datasets lack explicit grounding in a standardized threat model. Although some works reference the general APT lifecycle, they do not map their data collection to any formalized adversarial framework. Only two datasets utilize the MITRE ATT&CK framework: a dataset representing binary vectors of TTPs and IoCs to classify Android malware families [8], and a dataset mapping malware to MITRE tactics and techniques based solely on static features [7]*.* These datasets, while MITRE-aligned, do not incorporate runtime behavior and cannot simulate full APT progression.

The DEFEAT datasets are designed using both static and dynamic features mapped to MITRE tactics, enabling classification of APT stages, Device-level indicators, and App-level behavioral deviations. This design offers a more realistic simulation of APT progression and makes DEFEAT more directly applicable to operational threat-hunting and behavioral detection systems.

#### 3.1.4 Multi-stage APT coverage

Many existing datasets capture only one stage of attack activity. For example: Initial compromise via phishing or malicious URLs [5,6], C&C operations via DNS logs [3,4], or unspecified or partial stages [2,[9], [10], [11]]. Only two datasets in the literature cover multiple MITRE ATT&CK stages [7,8], and even these do so in a limited and inconsistent way, selecting tactics based on availability rather than modelling an attack chain. In contrast, DEFEAT benchmarks APT stage-level behavior rather than specific threat actors, enabling detection models to learn common behavioral indicators that are generalizable across malware families and attack stages. The Resource Usage Dataset captures the path from Initial Compromise to Credential Access and then to Exfiltration, while the App Based Dataset follows the path from Initial Compromise to Privilege Escalation and then to Exfiltration. These attack paths reflect the most common stages reported in real world APT operations and provide a more accurate foundation for analysing multi-stage APT attack.

#### 3.1.5 Online availability

The analysis shows that only two existing datasets capture APT activities across multiple attack stages [7,8]. Both of these datasets are still limited because they use only static features. As a result, they do not describe how APT attacks change their behavior over time on the device. In this study, the DEFEAT datasets include static features, such as permissions, sensors, and services; together with dynamic features of resource usage and traffic that are observed across several stages of the APT life cycle. Because the datasets are publicly available, other researchers can download them, repeat the experiments, and compare different detection methods under the same conditions. The same format also makes it practical to feed the data into machine learning experiments and security tools, which is important for everyday research work and for reproducible security studies.

The DEFEAT datasets are meant to be a more comprehensive and realistic choice for Android APT research. They follow the MITRE framework, combine static and dynamic views of the device, and span several stages of the APT attack. Taken together, this gives a clearer picture of attacker behavior and a common basis for evaluating detection methods.

## 4 Experimental Design, Materials and Methods

**Fig. 2:**
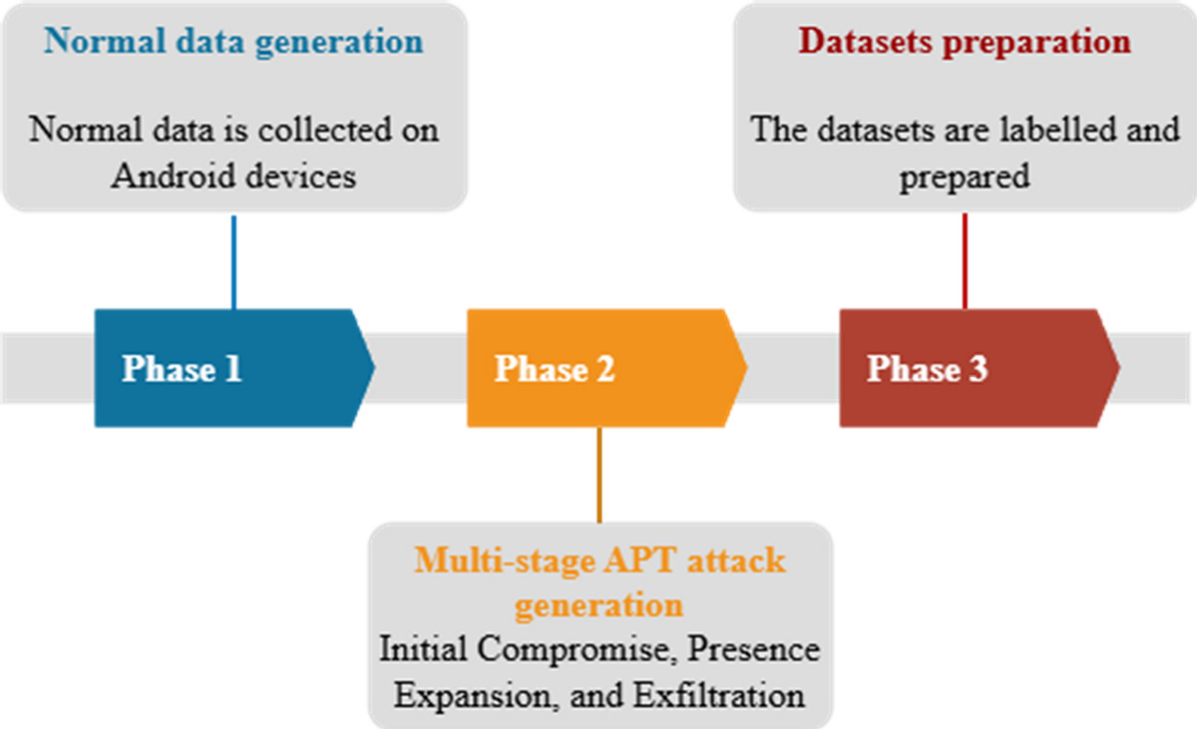
Methodology for collecting labelled DEFEAT datasets.

This section introduces the DEFEAT datasets. The datasets were created to fill gaps in existing Android APT research and to provide benchmark data for testing multi-stage APT detection models. The datasets include two types of features. The first type is dynamic resource usage features such as CPU usage, RAM usage, battery drain, RX, TX, and total traffic. The second type is app level features such as permissions, sensors, and services. The dataset generation follows three phases, as shown in Fig. 2. First, normal baseline data is collected. Second, a multi-stage APT attack is simulated on Android devices. Finally, the dataset is prepared for modelling and evaluation.

In order to evaluate detection performance, two testing strategies are used, cross-validation testing and supplied set testing. In the supplied set test, the datasets are divided into three subsets to measure performance on unseen data. In the supplied set test, the datasets are divided into three subsets: 64% was used to train and build the detection model, 16% for internal testing (validation), and 20% for unseen testing, simulating the model’s capability to detect previously unseen multi-stage APT activities.

This testing approach is essential, where detection models must detect new or stealthy APT activity that was not present in the training data. Accordingly, DEFEAT datasets are split into training, internal testing, and unseen testing subsets to support robust and realistic evaluation.

### 4.1 Network setup for data collection

**Fig. 3:**
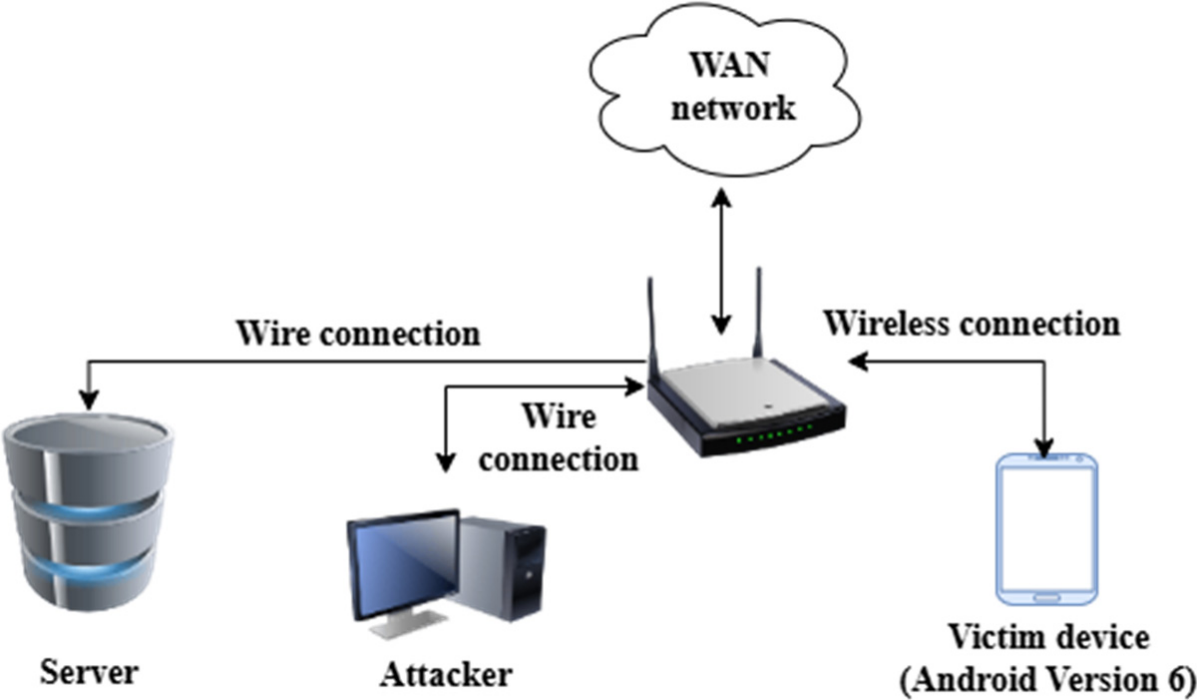
Network setup for data collection.

As shown in Fig. 3, the network testbed used for data collection was built around four main components: an Android device running version 6 (Marshmallow) acting as the user or victim, a Kali Linux machine representing the attacker, a wireless Cisco router, and a CentOS 7 server responsible for storing the collected data. The Android device connected to the router over Wi‑Fi, while both the Kali machine and the server were linked through Ethernet.

In order to make sure the data collected was accurate and dependable, both the client and server were configured. On the client side, the Android device ran a custom app collector called DEFENSE, which continuously captured resource‑usage and app‑based behavioral features and send the data to the server. Although Android 6 is an older release, the dataset is not intended to model Android-version-specific APIs or permission mechanisms. Instead, it focuses on fundamental device-level and apps-based behavioral features, that remain consistent across Android versions. These features are governed by the Linux kernel and application execution model, which have remained conceptually stable in modern Android releases. Consequently, the collected data reflect attacker-induced behavioral deviations rather than OS-version-specific indicators. Using Android 6 enables comprehensive, non-rooted access to these behavioral features, allowing accurate ground-truth collection that is increasingly difficult on newer versions due to restrictive privacy controls. This makes the dataset particularly valuable for developing and benchmarking behavior-based and risk-aware detection models that aim to generalize across Android versions rather than depend on OS-specific features.

On the server side, a CentOS 7 machine hosted a MySQL database used to store, organize, and manage all recorded data for further analysis.

### 4.2 Normal data generation

Several software can be used to create typical behavior in Android devices. A common practice is to use commercial tools that emulate user activities in the device. Although such tools are effective, they usually do not capture how people actually use their phones in everyday life and can lack the naturalistic uncertainty and variability present in real user activity. This can lead to data that appears artificial or biased, reducing its value for building reliable detection models. Another alternative is to utilize data from real-world networks which reflect real user behaviors and environmental conditions.

Based on these considerations, this research collected normal behavioral data from a real-world laboratory environment at Universiti Sains Malaysia (USM). The normal dataset includes behavior data from 40 high rated apps, as summarized in Table 8. Each app was executed for 10 minutes to simulate normal device behavior [12].

**Table 8:** Applications and categories used in normal data collection.

Category	Apps name	Collection time	Date and time
Watch apps	Todo list	10 mins each app	Monday 12/02/2024.
Android Auto	Good FM Dramas		
Art and design	Canava: Design, Photo and video		
Auto and vehicle	Maxim: Bike Taxi, car and Auto		
Beauty	Beaty camera plus: Sweet Cam		
Books and references	Al Quran		
Business	Flyers, poster maker, Design		
Comics	WEBTOON		
Communications	Messenger and Chrome		
Dating	Sexy video call & sexy chat		
Education	Duolingo: Language Lessons		
Entertainment	DramaBox-Stream Drama shorts and Youtube		
Events	Easy Quran Mp3 Audio offline		
Food and drink	Kenangan Coffee		
Games	Crossmath-Math puzzle Games		
Google Cast	Facebook		
Health and fitness	Home Workout no equipment		
House and home	Alfred Camera: Home security		
Kids	Elmo loves ABCs		
Lifestyle	Lemon 8-komuniti lifestyle		
Maps and navigations	Setel:Fuel, parking, e-walet		
Parenting	Asianparent: Kehamilan &bayi		
Personalization	Fonts Keyboards themes & Emoji		
Shopping	IKEA shopping		
Social	Cherry talk- random video chat and Tiktok		
Sports	Live football tv HD		
Tools	QR code scanner		
Travel and local	My ride Malysia’s E-Hailing		
Video players and editors	Screen recorder- xrecorder		
Weather	Local weather forecast		
Libraries and demo	Update apps for android		
Medical	Countour diabetes		
Music and audio	Ringtone maker		
Photography	Hypic		
Productivity	Cam scanner		
News and magazines	Podcast player		
Finance	Moomoo		

These apps were selected from 37 categories and obtained from trusted sources such as Google Play. Data collection was conducted over one day, on Monday, 12/02/2024 [13], while the DEFENSE app collected and transmitted data every 3 seconds to a remote server. The collected data was stored in CSV format for further analysis. In order to ensure data integrity, background activity on the device was carefully monitored throughout the collection period to confirm the absence of any abnormal behavior. For normal data collection, a total of 6,532 instances were recorded by observing the behavior of 40 legitimate apps.

### 4.3 Multi-stage APT generation

Generating multi-stage APT consists of three steps including Android malware apps identification, common APT stages determination, research assumptions, and the simulation process.

#### 4.3.1 Android malware identification

This section presents the identification of 36 Android malware samples listed in MITRE framework to simulate multi-stage APT. MITRE is a widely used cybersecurity framework adopted by researchers and analysts to study APT activities and to design countermeasures for both computer devices and mobile devices [14]. These 36 malware apps were selected for several reasons. First, the malware samples were implemented in multiple stages, as documented in the MITRE ATT&CK framework, which closely reflects the structure of real multi-stage APTs. Second, the behavior exhibited by these samples may resemble that of more recent malware variants. For example, attackers released four increasingly sophisticated versions of ZooPark [3], evolving from simple data-stealing tools into fully featured spyware, with each version built upon the previous one. Third, using well-documented and publicly available malware samples ensures reproducibility and transparency, allowing other researchers to replicate and validate the experimental results.

These identifies malware apps were downloaded from Android malware dataset (CIC-AndMal2017) [15] and GitHub repositories [16]. These apps are categorized into their known malware families including Adware, SMS malware, Backdoors, and Spyware (see Table 9).

**Table 9:** Abnormal malware applications used in the experiment.

Category	Family	App name
Adware	Gooligan (6)	Best wallpapers, memory booster, crazy motor, Cargame, HTM5 games, and smarttouch.
	Kemog (5)	Shareit, 2048kg, privacy guard, magic treasure, and sex academy.
	Shuanet (3)	Airdemon, Ninja turtles flapy, and Wild blackjack.
SMS malware	FakeInst (5)	Egypt 3d, Dragon Fighter 3d, Indian game, Photo Wonder, and Zalo.
Backdoor	Dendroid	Dendroid
Others	Exodus	Smartphone
	Anubis (3)	Borsa dovis Takip, nobetci eczane, and Doviz.
	Henbox	Backup
	Stealth Mango	google updater
	Zoopark (2)	(All in One) and Iranian app
	Bouncing Golf	Kik
	Moonkle	Google
	Riltok(3)	Youla, Aviasales, and Gumtree
	Xloader	Sex kr porn
	Joker/Bread	display camera
	Clipper	Metamask

The malware samples used in this study are not intended to represent specific nation-state threat actors. Instead, they are employed as behavioral proxies to simulate common APT stage characteristics on Android devices. Although some samples belong to adware or SMS-based malware families, they exhibit behaviors that overlap with documented APT tactics, such as establishing background communication channels, maintaining persistent execution, accessing sensitive resources, and exfiltrating data. Accordingly, the objective of the dataset is to benchmark stage-level behavioral patterns, such as stealthy execution during Initial Compromise and increased outbound communication during Exfiltration, rather than to attribute activity to a particular threat group.

These malware apps were validated using VirusTotal [17], a widely recognized threat intelligence platform that provides comprehensive security reports on files, URLs, and IPs. VirusTotal aggregates data from multiple antivirus engines and other security tools, making it a powerful platform for threat analysis. It scans each APK file to determine whether it is malicious. In order to validate malware apps are malicious, each APK file was uploaded to VirusTotal and analyzed. As illustrated in Table 10, the analysis process showed that several families are explicitly classified as SMS trojans, aggressive adware, spyware, and banking trojans this means that both the Threat category and family labels clearly point to how they behave after installation. For example, FakeInst1/2/3/8 and Joker appear with SMS-related tags such as trojansms, smsreg or smssend, which are used for malware that silently sends or reads SMS and registers the victim to premium services in the background. Gooligan, Kemoge and Shuanet variants are tagged with combinations like hiddenads, adware, drop-per, ztorg, ginmaster etc., and prior analyses show that these families often exploit root, hide as system apps, and continuously download and install new payloads without user interaction.

**Table 10:** Verification of Android malware samples using VirusTotal.

Group ID	Behavioral group	Families	Typical virus total labels
Group1	SMS-abusing malware (SMS Trojans)	FakeInst1/2/3/8, joker.apk	trojan, pua, adware, smskey, smsreg, smspay, trojansms, smssend.
Group2	Banking Trojans (credential & OTP theft)	Anubis1/3/4, Riltok1/2/3, Xloader1	trojan, downloader, dropper, banker, bankbot, riltok, wroba.
Group3	Spyware / cyber-espionage (APT-style mobile surveillance)	bouncinggolf.apk HenBox1.apk, Monokle1.apk, Zoopark1/2.apk, StealthMango1.apk	trojan, spyware, spyagent, domestickitten, henbox, monokle, zoopark, infostealer, apaspy.
Group4	Aggressive adware & auto-root droppers (repackaged apps)	Gooligan1/2/3/4/6/7/9, Gplayed.apk, Kemoge3/6/7/9/10, Shuanet1/7/10	trojan, adware, downloader, dropper, hiddenads, airpush, allad, ztorg, xinyinhe, ginmaster, kemoge, oveead, pluginloader.
Group5	Specialised trojans / loaders & info-stealers	clipper.apk, shuanet1.apk	trojan, clipper, pluginloader.

The spyware families (BouncingGolf, HenBox, Monokle, Zoopark, StealthMango) are documented mobile APT tools that, once installed, stay for long periods and quietly collect SMS, calls, microphone audio, location and other sensitive data. Finally, Anubis, Riltok and Xloader are well-known Android banking trojans that overlay banking apps and can intercept SMS one-time passwords or other financial data.

#### 4.3.2 Common APT stages

**Fig. 4:**
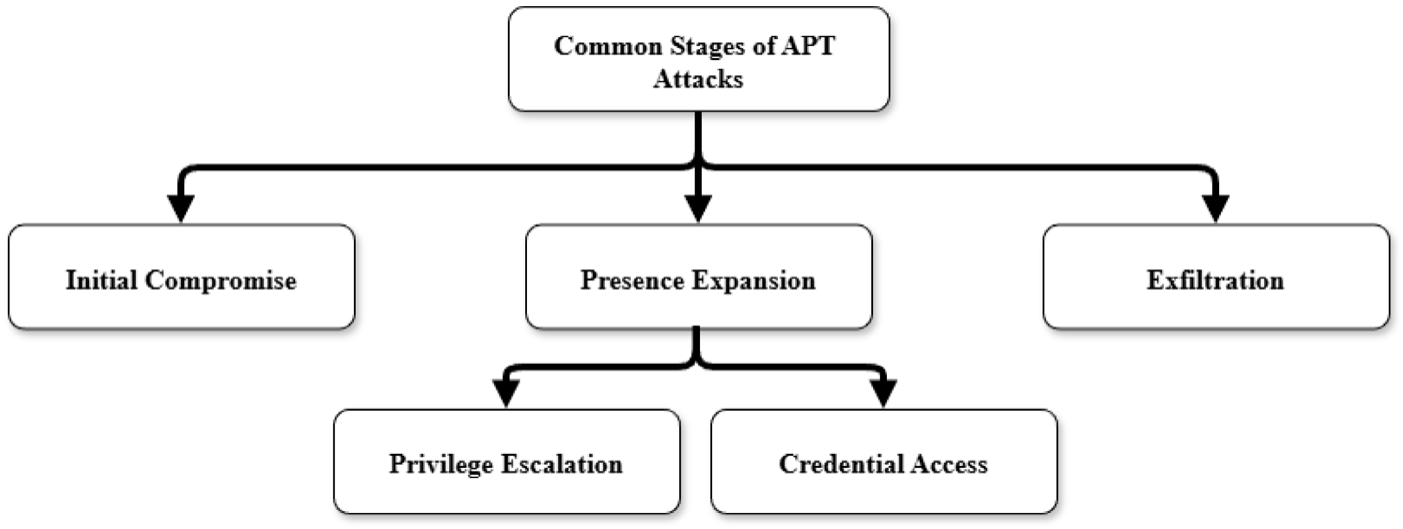
Common stages of APT attacks.

Although Android malware apps were implanted in different stages (Tactics) as documented in MITRE framework, generalizing the attack stages is critical to detect multi-stage APT. These stages represent the attack life cycle which starts with Initial Compromise and Ends with Exfiltration stages. Based on the analysis (Fig. 4), three common stages observed in real world observations include Initial Compromise, Presence Expansion, and Exfiltration [2].

In Initial Compromise, the attackers are trying to compromise the targeted devices using various attack vectors such as app repackaging. In Presence Expansion, Once the attackers successfully compromise the targeted device, they typically work to broaden their access and collect sensitive information such as usernames and passwords. With these privileges, they can reach valuable business data and create persistent backdoors, allowing them to maintain long-term, covert access. In the final stage, attackers extract the stolen information from the compromised devices without detection.

Based on these findings, this research identifies four main stages: Initial Compromise, Privilege Escalation, Credential Access, and Exfiltration. These stages are critical because an attacker must first gain access to the targeted device, then escalate privileges and obtain credentials, which enable lateral movement across the network to compromise additional systems [18]. The final stage involves exfiltrating the sensitive information gathered during the attack.

#### 4.3.3 Research assumption of the APT scenario

This section outlines the research assumptions used to simulate multi-stage APT on Android devices. a) Attacker scenarios

The simulated attacks in this study focus on observable behavioral effects on the device and apps rather than simulating a specific threat actor. This level of abstraction enables consistent modelling of APT stages across heterogeneous malware families while preserving the essential behavioral indicators relevant for detection. Accordingly, the simulated behavior is organized into a stage-based sequence aligned with the established MITRE framework.

• Initial Compromise: In this stage, the attacker gets a first entry point by tricking the victim into installing a malicious app, often through spear phishing, watering-hole attacks, or other social engineering. This study does not focus on how the app is delivered. Instead, it focuses on what happens after installation by monitoring and recording device and app behavior once the malware is present.
• Presence Expansion: After gaining access, the attacker tries to strengthen control over the device and begin collecting valuable information. The study tracks device behavior by observing which permissions, sensors, and services the malicious apps try to use. Common actions are to include privilege escalation to obtain higher access and credential access to collect login information such as email accounts and passwords.
• Exfiltration: In the final stage, the attacker sends the collected data out of the device to external servers, often through command-and-control channels or other communication methods. This study records the behaviors linked to data exfiltration performed by malicious apps through a command-and-control channel.

b) User (Victim) scenarios

This research assumes that users are relying on older Android devices and may not be fully aware of the risks of installing apps from third-party sources. Because of this limited security knowledge, they might unintentionally download untrusted or malicious applications, which increases their vulnerability to these types of threats.

#### 4.3.4 Simulation process

After android malware apps, common APT stages, and research assumptions are identified. This section presents the simulation process of multi-stage APT on Android devices. Multi-stage attacks could not be conducted on the real USM network due to the potential risk of affecting network performance and user devices. Android malware can spread and compromise other devices within poorly protected environments. As such, the proposed multi-stage APT datasets were generated within an isolated and controlled environment.

In order to simulate realistic multi-stage attack, a reverse TCP payload (e.g., android/meterpreter/reverse_tcp) was injected into each malware app using the msfvenom tool within the Metasploit framework in Kali Linux. The generated payload was hosted on an Apache server and linked to a listener created in *msfconsole*. This setup let the malware run safely inside a controlled environment, where its actions could be observed and recorded in detail. The experiment followed the same sequence of stages seen in real APT operations: Initial Compromise, Presence Expansion, and Data Exfiltration [2]. These stages are critical because an attacker typically starts by getting a foothold on the device, then attempts to increase privileges and gather credentials. With those credentials, the attacker can move laterally across the network and begin targeting other systems [18]. The final stage involves exfiltrating sensitive information from targeted systems. Table 11 shows the performed commands in simulating each attack stage (Initial Compromise, Presence Expansion, and Exfiltration) to establish the connection between the client (Android device) and C&C server.

**Table 11:** Performed commands on the client (victim) and C&C server side.

S. no.	Activity	Device	Description
**1.**	Reverse TCP payload injection	Client (Android device)	sudo msfvenom -x **app-name.apk** -p android/meterpreter/reverse_tcp LHOST= **server-IP-address LPORT=server-port-number -o output.apk**
**2.**	Establish the connection	C&C Server	• sudo systemctl start apache2 • msfconsole • use exploit/multi/handler • set payload android/meterpreter/reverse_tcp • set LHOST **server-IP-address** • exploit
**3.**	Initial Compromise	Client (Android device)	Collecting data without triggering any activity
**4.>**	Presence Expansion	Client (Android device)	Escalate privileges and gather credentials
**5.**	Exfiltration	Client (Android device)	Exfiltrate sensitive data to C&C server

The resulting output comprised two datasets across two attack path scenarios: The first dataset leverages the attack path of Initial Compromise → Credential Access → Exfiltration, based on resource usage features. While the second dataset follows the attack path of Initial Compromise → Privilege Escalation → Exfiltration, using app permission, sensor, and service features.

**Fig. 5:**
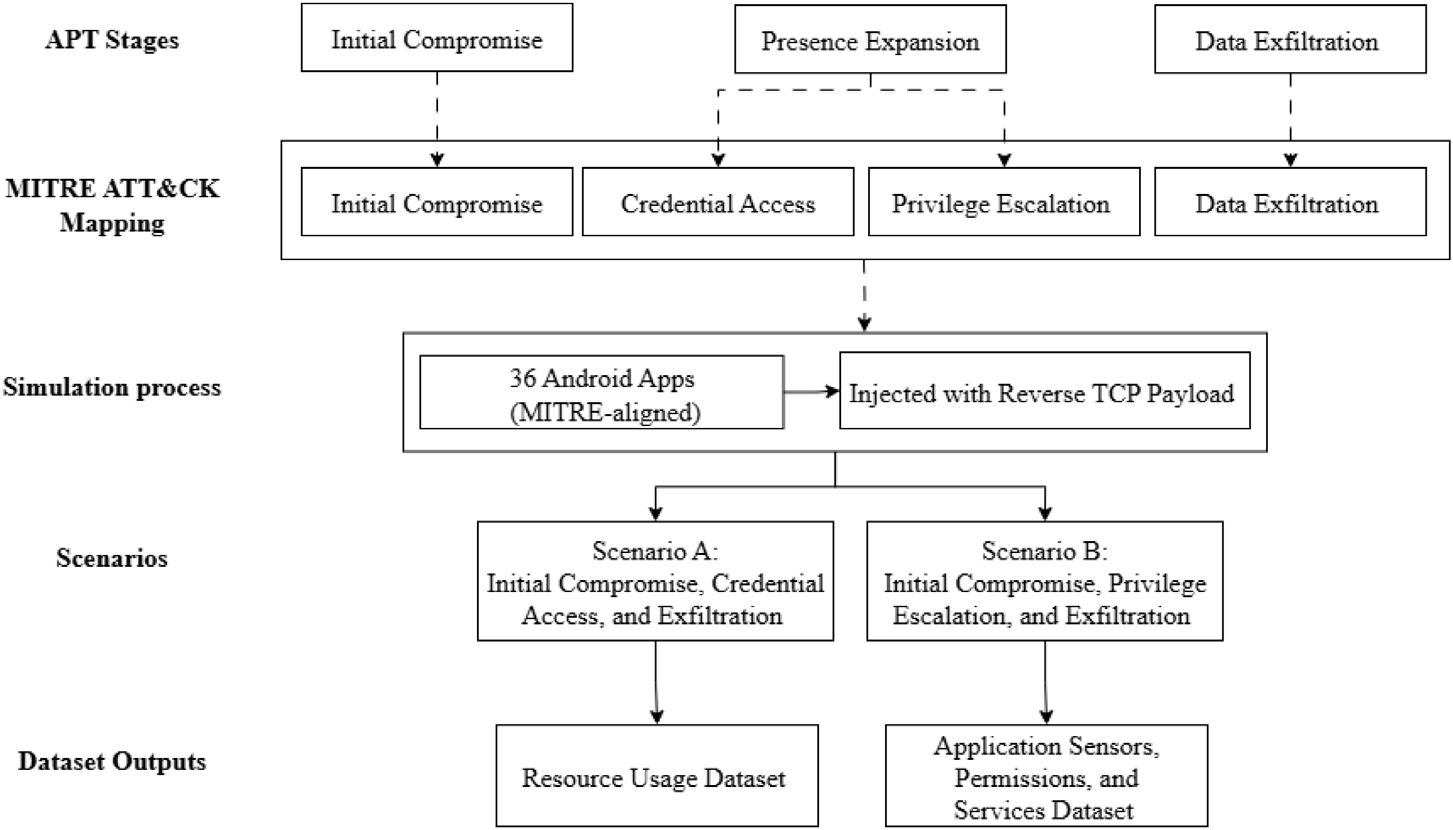
Workflow simulation of APT stages aligned with MITRE ATT&CK

Fig. 5 illustrates how generalized APT stages are mapped to MITRE ATT&CK framework, Android malware samples were simulated, and the resulting datasets. Both scenarios generate both datasets concurrently. This workflow was implemented as part of the study and is based on ATT&CK-aligned simulation of multi-stage Android threats.

Each abnormal app was executed for 10 minutes, and the device was reset between each execution to ensure a clean testbed environment. This execution time is sufficient to capture behavioral changes across stages, as supported by prior study [12]. While 10 minutes is sufficient to capture the active behavioral manifestations of these specific attack stages, it does not capture the dormancy periods typical of long-term APT campaigns. The DEFENSE app continuously monitored and transmitted behavioral data every 3 seconds. As illustrated in Table 12, this research collect multi-stage APT datasets over three consecutive days, one day for each stage as followed by the prior research [13] that simulated multi-stage APT dataset on computer devices.

**Table 12:** Collection details of each APT stage.

APT stages	No. of abnormal apps	Collection time	Date and time
Initial Compromise	36	10 mins every app	Tuesday 13/02/2024
Presence Expansion	36	10 mins every app	Wednesday 14/02/2024
Exfiltration	36	10 mins every app	Thursday 15/02/2024

This variety of malicious apps implemented during multiple attack stages was designed to offer broad and realistic coverage of potential multi-stage APT activities. Capturing this range of behavior is important for building an effective detection approach. During abnormal data collection, a total of 6,209, 6,229, and 6,301 instances were recorded by observing the behavior of 36 malicious apps across the Initial Compromise, Presence Expansion, and Exfiltration stages, respectively.

### 4.4 DEFEAT datasets preparation

This section presents the preparation of the DEFEAT datasets: (1) resource usage and (2) app permissions, sensors, and services datasets, which were generated in previous sections. The resource usage dataset is prepared to include the 8 selected features (see Table 1), excluding any non-qualified feature values. Similarly, the app permissions, sensors, and services dataset are prepared to include 108 features (see Table 2). Both datasets undergo preprocessing steps before they are ready to be used with classification models.

#### 4.4.1 Labelling

The dataset is an important part in the evaluation of the detection systems which depend on labelled data. The app collector (DEFENSE) tool has been used to capture and send the data into the server which can be saved as a comma-separated values (CSV) file. The collected datasets are labelled by adding a class label (normal, abnormal) to each record as follows; the CSV file is used to record all data of both activities. Then, each activity row is deterministically labelled based on the author’s knowledge of the attacks’ nature, simulation time, and the nature of triggered features.

#### 4.4.2 Normalization

Normalization is a necessary process to complete the preparation of the datasets before applying any classification models. Non-numeric attributes are replaced with distinct numeric values (nominal values) to enable proper handling and improve classification performance. Moreover, several features exhibit large value ranges, which could skew the model’s results. Therefore, normalization is applied to ensure that all features contribute equally to the learning process.

#### 4.4.3 Balancing

The final step in dataset preparation involves balancing the two class labels: normal and abnormal. The Synthetic Minority Oversampling Technique (SMOTE) is one of the most widely used methods for addressing class imbalance in the literature [19,20]. SMOTE balances the dataset by synthetically generating new instances based on the known distribution of existing data. This process improves the generalization capacity of any applied classifier.

In this study, normal instances were increased in the training dataset using SMOTE to simulate real-world scenarios, as multi-stage APT activities rarely occur compared to normal activities. As shown in Table 13, the SMOTE technique successfully balanced the dataset between the two class labels, resulting in 52% representation for the normal class using Python. As a result of the dataset preparation process, two balanced, normalized, and labelled DEFEAT datasets were created. These datasets are designed for training and testing detection systems aimed at detecting multi-stage APT attacks on Android devices. After normalization, all features were converted into numeric data types, making them ready for use with machine learning classifiers. Since the targeted threats are multi-stage APT, the class label is treated as a nominal attribute with two possible values (normal and abnormal). Thus, the datasets are considered binary classification datasets.

**Table 13:** Specifications of the generated DEFEAT-based training and testing datasets.

Dataset	Total data	Training dataset – before resampling (80%)		Training dataset - after resampling		Test dataset (20%)	
		Normal	Attack	Normal	Attack	Normal	Attack
Initial Compromise	12,741	5225	4967	5380	4967	1307	1242
Presence Expansion	12,761	5225	4983	5398	4983	1307	1246
Exfiltration	12,833	5225	5041	5460	5041	1307	1260

## 5 DEFEAT datasets validation and evaluation

In order to verify the worthiness of the proposed datasets, their characteristics against the five requirements of the good dataset are analysed. In addition to fulfilling these five quality criteria, these datasets evaluated using six different classifiers to demonstrate their applicability with existing detection models.

### 5.1 DEFEAT datasets validation

This section analyses the validation of the proposed DEFEAT datasets against the five requirements of a good dataset [19]. The analysis below validates the extent to which the proposed datasets satisfy these criteria.

#### 5.1.1 Realistic data

This requirement is essential for capturing the user interactions and responses on Android devices. In this study, it was satisfied by collecting real behavioral data from a physical Android device, including: (1) resource-usage, and (2) app permissions, sensors, and services. All data was gathered from a physical device, rather than using emulators or sandbox environments. The collection process spanned four days, allowing the datasets to reflect common multi-stage APT attack paths without applying sampling techniques that might remove important information. Normal behavior was continuously recorded using the DEFENSE app, while the multi-stage APT scenarios were carried out within an isolated network environment to ensure that malware did not propagate outside the controlled setup.

#### 5.1.2 Scenarios diversity

Multi-stage APT attacks are triggered over multiple stages. In this study, the attack path was simulated using three main stages, Initial Compromise, Presence Expansion, and Exfiltration. The DEFEAT datasets were collected over four working days through controlled experiments on a real Android device, where both device and application behavior were monitored. The data collection followed a clear schedule. On the first day, normal baseline activity was recorded using 40 high rated benign apps. Over the next three days, the multi-stage APT path was simulated using 36 malicious apps, with each stage carried out based on its description in the MITRE framework. This setup, which combines multiple stages and both benign and malicious apps, helps capture a wide range of behaviors needed to support effective detection.

#### 5.1.3 Completed and correct labelling

Correct labelling is important when preparing a dataset for the detection process. In this study, every record, whether normal or abnormal, was labelled manually. The labels were assigned on the basis of the author knowledge, simulation time, and the monitored feature behavior. For both datasets, normal behavior was labelled as Class 0 and abnormal behavior as Class 1. This consistent method labelling supports model training by differentiate clearly between normal and attack activity.

Each label corresponds to a single recorded instance, where every row represents a snapshot of the device’s behavior at that moment. This ensured that normal entries truly came from everyday, harmless use, while all abnormal entries were taken strictly from periods when the simulated attacks were running. To make sure the labels were correct, a number of samples from both classes were checked manually. These spot checks helped verify that the recorded behavior matched what would reasonably be expected in either a normal or an attack scenario.

#### 5.1.4 Sufficient size

Although having a large dataset is always beneficial, what matters most is making sure that each class is represented with reasonable instances. When one class dominates the dataset, the classifier tends to learn it, which leads to biased learning and poor performance on the minority class [19]. A more balanced distribution gives the model a fair chance to learn the behavior of both normal and abnormal activities.

While both oversampling and under sampling are commonly used to address imbalance datasets, this study avoided under sampling to prevent losing useful information. Instead, this study used SMOTE, which generates new samples for the minority class by learning from the patterns already present. This method helped to achieve a more even distribution without losing any of the original data. As a result, the training set became more representative and helped the classifier perform better when dealing with new, unseen behavior.

#### 5.1.5 Representative features

This requirement highlights to ensure that the features included in the DEFEAT datasets are effective for validating security models. As discussed in the Section 4, two kinds of datasets were created: a resource-usage dataset with (8) features as showed in the Table 1 and an app-based dataset with 108 features capturing sensors, permissions, and services as seen in the Table 2. Each feature was selected based on their effectiveness to detect traditional and APT attacks. Any feature that lacked relevance or showed inconsistent behavior during preliminary checks was removed to avoid adding noise or misleading patterns. After finalizing the feature sets, several machine learning classifiers were applied to test whether these features could be effective in detecting APT activities.

Two different testing approaches (cross-validation testing and supplied set testing) were applied to evaluate the robustness of the features. During early checks, any feature that appeared irrelevant or behaved inconsistently was removed to avoid adding noise or misleading patterns. After the final feature sets were confirmed, multiple machine-learning classifiers were trained and tested to verify that these features can support APT detection in practice. To assess how stable the results are, two evaluation settings were used: cross-validation (to measure performance across repeated data splits) and a supplied test set (to test performance on unseen data). These approaches were also intended to verify the applicability of the datasets for multi-stage APT detection.

### 5.2 SHAP analysis

In this section, SHAP analysis is performed to both datasets, resource-usage and application-based datasets, to show the contribution of each feature to detect APT activities across the three stages using SHAP beeswarm plot.

#### 5.2.1 SHAP analysis for resource usage features

**Fig. 6:**
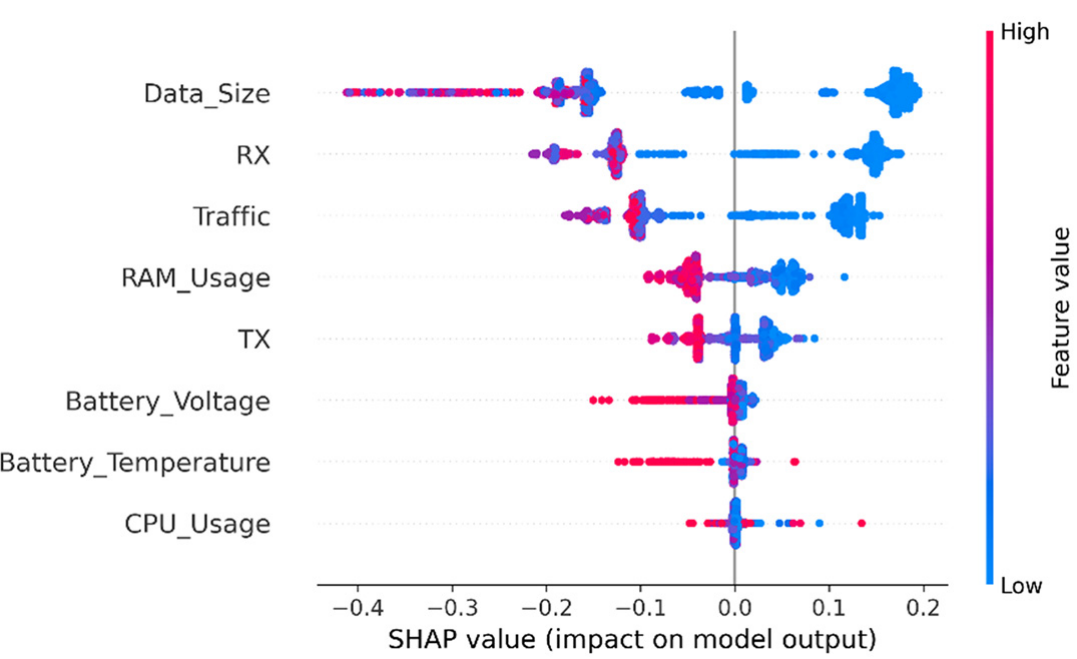
SHAP analysis for resource usage - Initial Compromise dataset

**Fig. 7:**
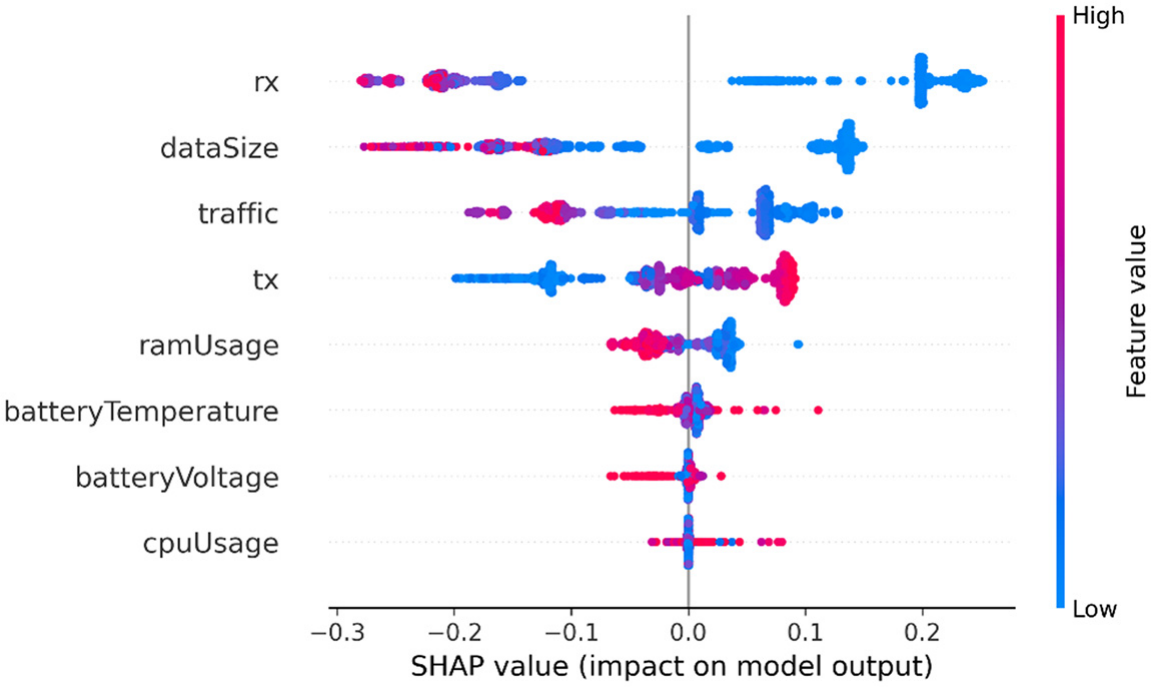
SHAP analysis for resource usage - Credential Access dataset

**Fig. 8:**
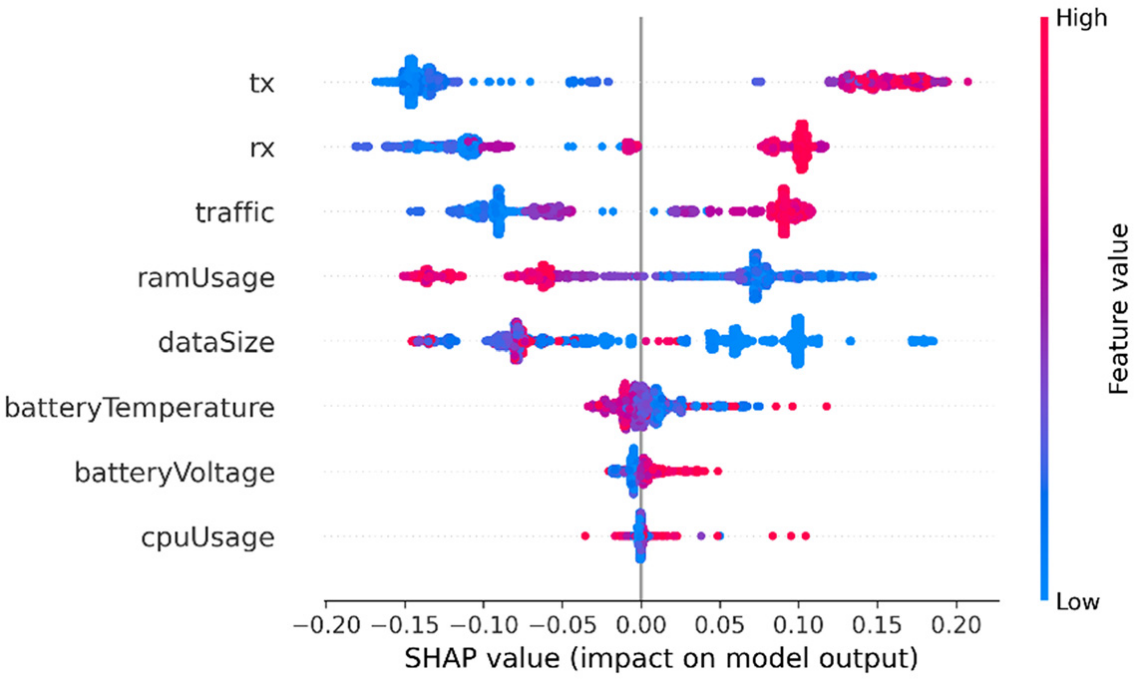
SHAP analysis for resource usage - Exfiltration dataset

As illustrated in Fig. 6, Fig. 7, Fig. 8, SHAP beeswarm plot is used to identify the most contributing features to detect APT across three stages: Initial Compromise, Credential Access, and Exfiltration. Each dot represents an instance, positioned on the *x*-axis according to the SHAP value, with color indicating the feature’s original value, red for high values and blue for low values.

The SHAP analysis of the Initial Compromise dataset (see Fig. 6), shows that most features exhibit a negative SHAP impact when their values are high (i.e., red points appear predominantly on the left side of the SHAP axis). This means that high feature values generally push the model toward detecting the “normal” class. Conversely, low feature values (blue points) often appear on the right side of the plot, meaning they push the model toward detecting the “attack” class. This matches typical APT behavior during the Initial Compromise stage as the attackers try to stay hidden and avoid doing any activities on the device. So, the results show low resource-usage values that act as an early warning sign, while higher values often look more like normal activity and therefore reduce the chance of predicting an attack. The SHAP analysis for the Credential Access dataset (Fig. 7) shows a different pattern from Initial Compromise. The attacker starts actively trying to obtain sensitive information such as account credentials. Thus the model shows TX (outbound traffic) is the strongest signal when TX is high (shown as red points on the positive side of the SHAP axis), it pushes predictions toward the “attack” class. This suggests that increased outbound communication is a key sign of credential-access activity in this stage of the dataset.

In contrast, some other features, like RX and total traffic, have SHAP values that are mixed or close to zero. This suggests that incoming traffic and overall network use stay fairly stable, so they don’t clearly separate normal behavior from malicious behavior at this stage. The same pattern appears for RAM usage, app data size, CPU usage, and battery-related features, their SHAP effects are mostly negative or near zero. In other words, when these values increase, the model is more likely to predict “normal,” which suggests the attacker is still trying to keep activity low and less noticeable at this point. Overall, the SHAP patterns highlight that the primary behavioral shift in the Credential Access stage is increased outbound data transmission, while most other device-level features still resemble normal activity. This aligns with the expected nature of credential-access malware, which typically extracts and sends sensitive information without yet causing heavy local resource usage.

Finally, the SHAP analysis of the Exfiltration dataset (see Fig. 8) reveals the strongest behavioral shift among the three APT stages. Unlike the Initial Compromise and Credential Access stages, where the attacker remains relatively quiet, exfiltration involves data transfer as the attacker sends stolen information outside the targeted device. This behavioral change is clearly reflected in the SHAP patterns.

In this stage, RX, TX, and overall Traffic all show strong positive SHAP contributions. In the figure, high values of these features (indicated by red points) appear predominantly on the right side of the SHAP axis. This means that high traffic activities push the model toward detecting the “attack” class. This pattern fits what usually happens during the exfiltration stage, where the malware’s main effort goes into exfiltrating stolen data from the targeted device. As a result, network activity becomes the clearest indicator of malicious behavior. In contrast, features like RAM usage, app data size, battery temperature or voltage, and CPU usage have only a minor impact. When these values increase, they tend to produce neutral or slightly negative SHAP effects, meaning they don’t clearly separate an exfiltration attempt from normal device activity. This shows that even at the final stage of the attack, the malware tries to avoid drawing attention by keeping its local resource usage low and focusing instead on its network operations. Overall, the SHAP results show that increased network activity is the dominant indicator of exfiltration, with RX, TX, and total Traffic emerging as the most reliable predictors for detecting this final and most active stage of the APT lifecycle.

#### 5.2.2 SHAP analysis for app features

For clarity and comparability across stages, only the top 20 features ranked by mean absolute SHAP value are displayed in each beeswarm plot. This focuses the interpretation on the most important permissions, sensors, and services that drive the model’s decisions, while less informative features remain in the long tail and are not shown clearly. In all SHAP beeswarm plots, each point represents one instance from the dataset. The horizontal axis shows the SHAP value of a feature which refers to how much that feature pushes the model output away from the baseline prediction. Negative values on the left side move the prediction toward normal, while positive values on the right side move it toward attack. Features are sorted from top to bottom by their mean absolute SHAP value, so the top rows correspond to the globally most influential features. The colour of each point indicates the original feature value, with red indicating high values and blue indicating low values.

**Fig. 9:**
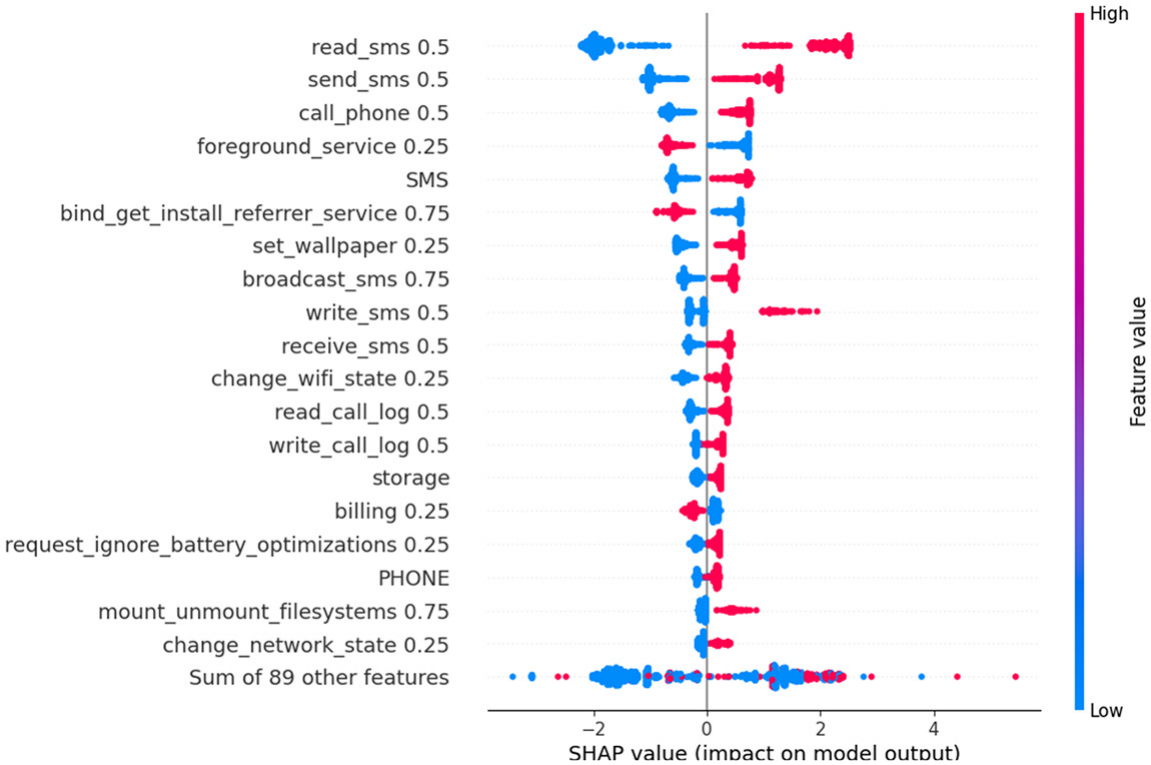
SHAP analysis for application-based - Initial Compromise dataset

The SHAP analysis of the Initial Compromise dataset as indicated in Fig. 9 shows that the SMS and telephony permissions like read_sms 0.5, send_sms 0.5, call_phone 0.5 and the SMS group have high values (red points) on the right side of the SHAP axis, while low values (blue points) lie mainly on the left. This means that activating these capabilities pushes the model toward the attack class. Additional SMS related features such as broadcast_sms 0.75, write_sms 0.5, receive_sms 0.5, together with read_call_log 0.5 and write_call_log 0.5, also contribute positively when enabled but with smaller SHAP scale, indicating that the malware is already preparing the SMS and call channel but with moderate tend in this stage.

In contrast, several features exhibit an inverse relationship with the attack class. For foreground_service 0.25 and bind_get_install_referrer_service 0.75, high values (red points) are concentrated on the left side of the SHAP axis and low values (blue points) on the right. This means that enabling these capabilities tends to pull the prediction toward the normal class, whereas their absence slightly increases the probability of an APT.

**Fig. 10:**
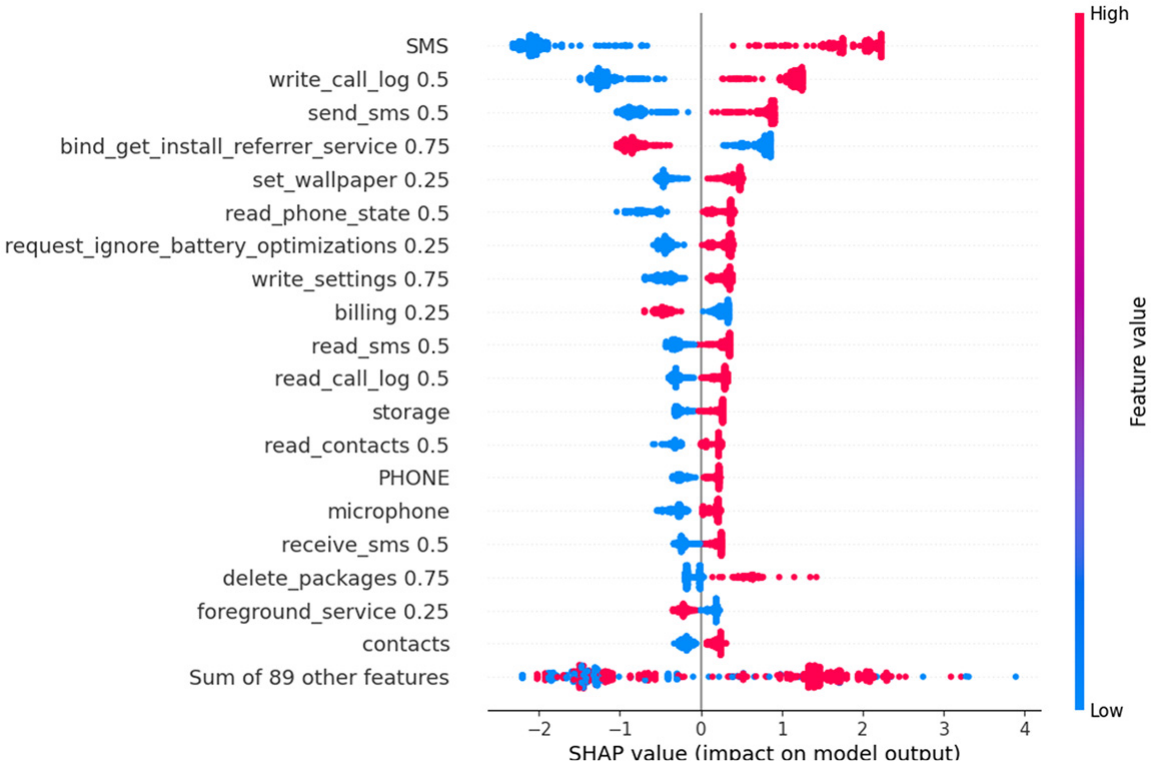
SHAP analysis for application-based – Privilege Escalation dataset

In the Privilege Escalation dataset (Fig. 10), the SHAP ranking is still dominated by the SMS and calls with the permissions like SMS, write_call_log 0.5 and send_sms 0.5 show high values mainly on the right of the axis and low values on the left, meaning that frequent SMS use and call-log push the model toward the attack class, while their absence supports normal behavior. System-level control features such as request_ignore_battery_optimizations 0.25, write_settings 0.75 and delete_packages 0.75 also have red points concentrated on the right, indicating that reduce power management, changing device settings and silently removing packages all increase the likelihood of an APT decision.

In contrast, bind_get_install_referrer_service 0.75, billing 0.25 and foreground_service 0.25 also exhibit an inverse pattern in this stage, high values are mostly on the left and low values on the right. This shows that active use of install-referrer API, in-app billing, and user-visible foreground services is more typical of benign apps, so their presence pulls predictions toward the normal class, whereas their absence in combination with offensive SMS and call behavior increases the probability of an APT. The microphone feature appears with moderate positive SHAP values (red points on the right), marking Stage 2 as the first point where a sensor becomes clearly involved. Overall, the Stage-2 pattern indicates that the attacker strengthens communication control, gains broader system privileges, and begins microphone-based sensing, which together characterizes the Privilege Escalation stage.

**Fig. 11:**
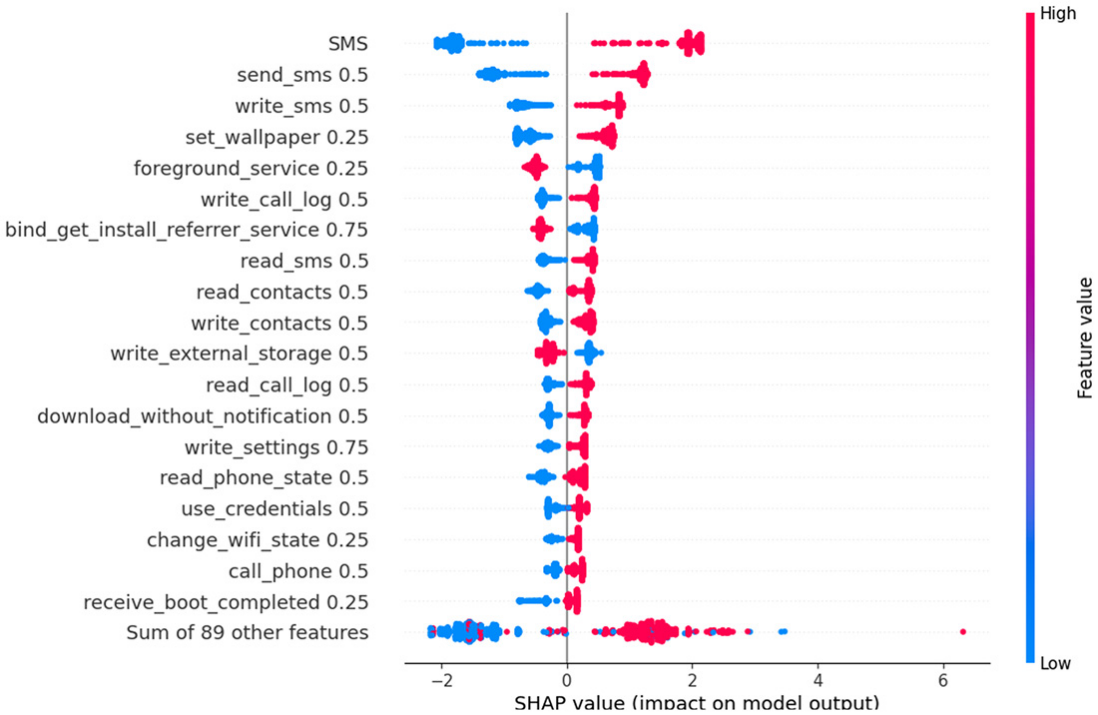
SHAP analysis for application-based - Exfiltration dataset

The SHAP analysis of the Exfiltration dataset (Fig. 11) shows the strongest concentration of high impact app features. The SMS, send_sms 0.5, write_sms 0.5, and read_sms 0.5 have high values clearly clustered on the right of the SHAP axis and low values on the left, confirming that intensive SMS manipulation is the main features of attack predictions in this stage.

Permissions related to contacts and calls also become dominant. High values of read_contacts 0.5, write_contacts 0.5, read_call_log 0.5, download_without_notification 0.5, use_credentials 0.5 and write_settings 0.75 lie mostly on the right, indicating that accessing or modifying contacts, inspecting call logs, performing silent downloads and using stored credentials are strongly associated with exfiltration behavior. Meanwhile, Persistence related features like receive_boot_completed 0.25 retains a positive SHAP impact, showing that the malware continues to auto start while data are being stolen.

An important observation is that write_external_storage 0.5 shows an inverse relationship with the attack class in the Exfiltration stage, high values appear predominantly on the left of the SHAP axis, while low values are more concentrated on the right. This indicates that intensive writing to external storage is more typical of benign apps, whereas the APT samples mainly exfiltrate data directly via SMS, contact access and network channels without leaving large files on shared storage. This pattern is consistent with APT and spyware campaigns that aim to minimize their on-disk footprint and avoid user-visible traces by streaming data out in memory or over the network rather than staging it on external storage. Meanwhile, As shown in Fig. 9, Fig. 10, Fig. 11, the top-ranked SHAP features are dominated by permissions with a weight of 0.5, corresponding to Android’s Dangerous level (for example, read_sms 0.5, send_sms 0.5, read_call_log 0.5, write_call_log 0.5, read_contacts 0.5, write_contacts 0.5, write_external_storage 0.5). This aligns with Android’s definition of Dangerous permissions as those that grant access to private user data or sensitive operations such as SMS, call logs, contacts, and external storage access. The relatively smaller contribution of normal (0.25) and very high-tier Signature/Privileged (0.75/1.0) permissions among the top features indicates that the APT attack mainly exploits realistic permissions that are powerful enough to expose sensitive content of the mobile device.

### 5.3 t-test statistical analysis of malware behavior across APT stages

The purpose of this analysis is not only to demonstrate behavioral variability across malware families, but also to confirm that each APT stage exhibits statistically distinct behavioral patterns. This supports the validity of stage-based APT modelling independent of the specific attack tools or communication payloads used. Accordingly, this section examines whether the collected resource-usage features capture intrinsic malware behavior and stage progression, or whether they merely capture the behavior associated with a common command-and-control payload. Although the same Meterpreter reverse TCP payload was employed to establish command-and-control communication throughout all simulated APT stages (Initial Compromise, Credential Access, and Exfiltration), the analysis is designed to assess whether meaningful behavioral distinctions remain observable beyond this shared communication channel.

**Fig. 12:**
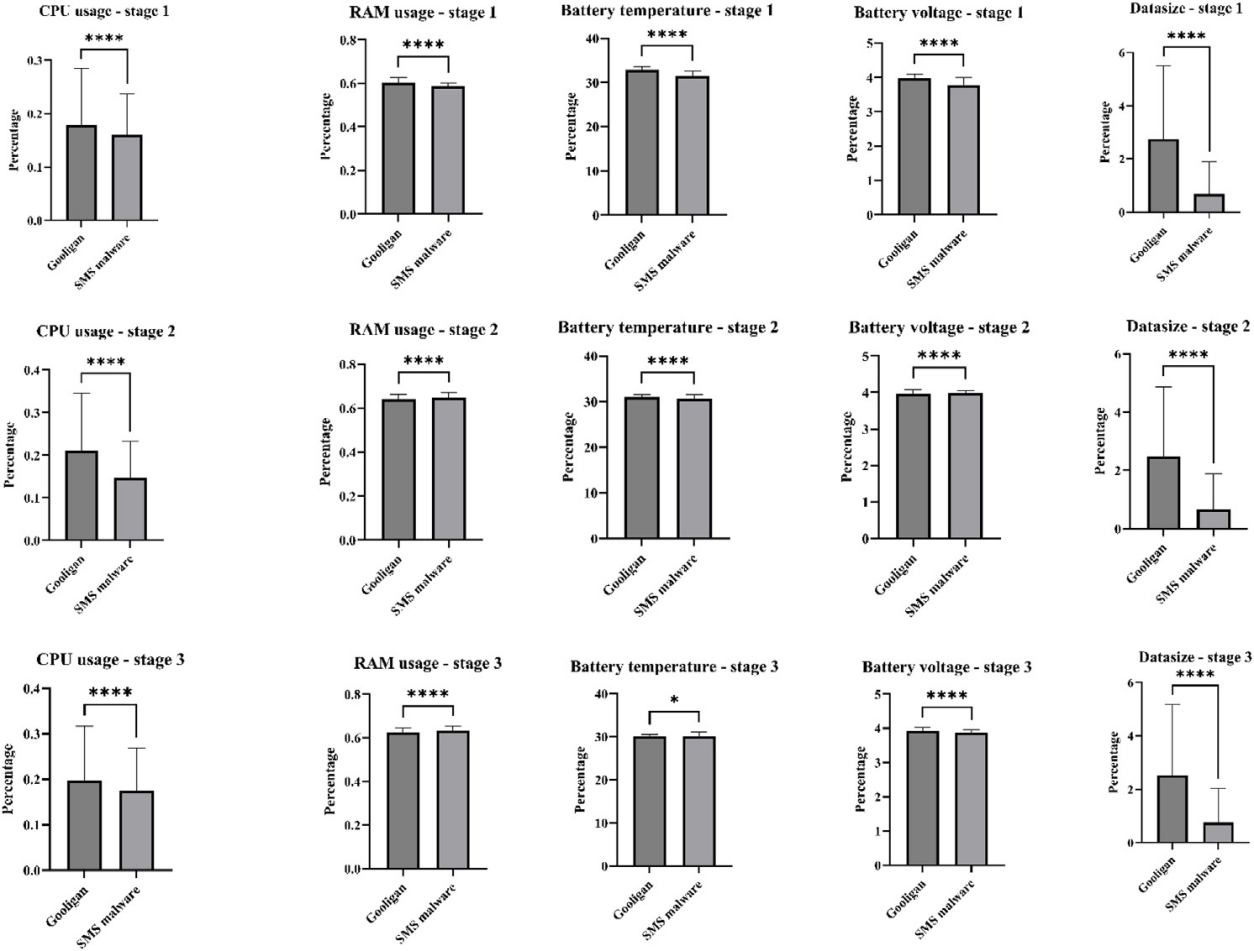
Test analysis for CPU, RAM, and Battery usage features of two malware families

**Fig. 13:**
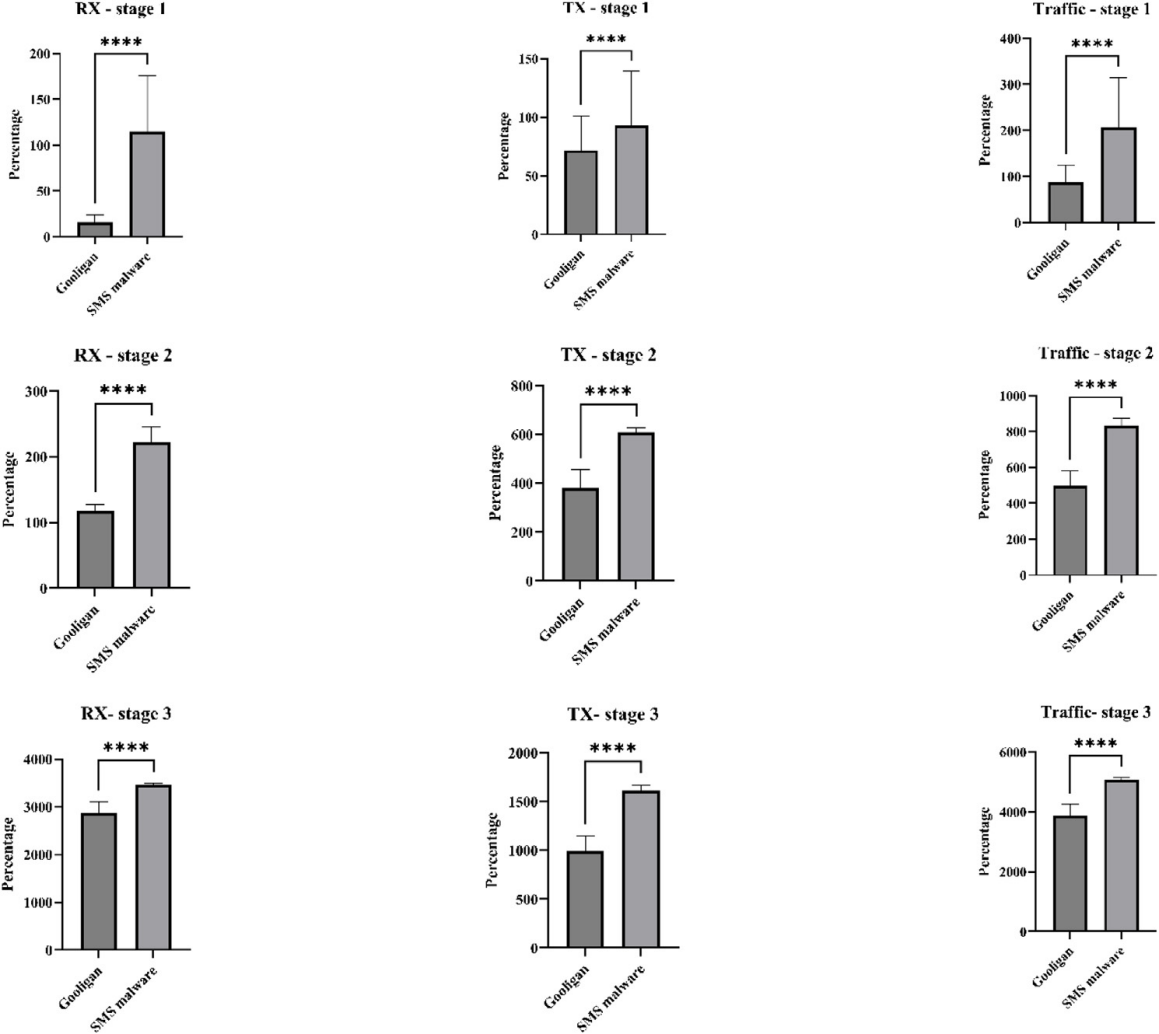
Test analysis for RX, TX, and traffic features of two malware families

To investigate this, a statistical t-test analysis was conducted on key resource-usage features, including CPU usage, RAM usage, battery voltage and temperature, application data size, RX, TX, and total network traffic. The analysis focused on representative samples from two distinct malware families, namely Gooligan and SMS-based malware, across all three APT stages. As illustrated in Fig. 12, Fig. 13, most features exhibit statistically significant differences between malware families (*p*<0.0001), despite the use of an identical payload configuration.

These results provide strong evidence that the collected behavioral features are not payload-driven. The Meterpreter payload primarily serves as a communication mechanism to enable remote interaction with the compromised device. In contrast, resource-consumption patterns are governed by the internal functionality, background processes, and operational goals of each malware family. Consequently, even when a common payload is used, different malware families generate distinct and measurable behavioral footprints. In addition, the results confirm that behaviors associated with later APT stages, particularly Exfiltration, are statistically distinguishable from those observed during Initial Compromise. This stage-level separation validates the dataset’s alignment with multi-stage APT behavior rather than malware-family-specific characteristics alone.

Moreover, the observed variability in features such as RX, TX, total traffic, CPU usage, and application data size reflects both malware-specific behavior and stage-specific objectives. During an earlier stage, malware tends to operate stealthily with limited resource usage, while later stages (Credential Access and Exfiltration) exhibit increased communication and data-transfer activity. The presence of wider error bars in several features further indicates natural variability arising from the heterogeneous nature of the malware samples, which differ substantially in functionality, execution flow, and resource demands.

Overall, this analysis confirms that the DEFEAT datasets capture meaningful, malware-specific, and stage-dependent behavioral characteristics rather than indicators of a shared payload.

### 5.4 DEFEAT datasets evaluation

In this section, six AI classification techniques are applied to evaluate the proposed DEFEAT datasets using two different testing approaches. The first approach is cross-validation testing, where the classifiers are trained on a portion of the dataset and tested on the remaining portion. The second approach is the supplied test set, where classifiers are trained on a training dataset and tested on previously unseen dataset. These classifiers are used in their standard forms without any enhancement or parameter tuning as the main aim is to demonstrate the reliability and trustworthiness of the proposed datasets for evaluating multi-stage APT detection. Additionally, this evaluation aims to confirm that the proposed resource usage features and app permissions, sensors, and services features are capable of distinguishing between multi-stage APT activities and normal activities.

Three evaluation metrics are used to evaluate the effectiveness of the datasets and their features: detection accuracy, FPR, and FNR. Detection accuracy measures the percentage of correctly classified normal and attack records, and is calculated as shown in Eq. (1):

(1) $$Accuracy=\frac{TP\;+\;TN}{TP\;+\;TN\;+\;FP\;+\;FN}*100\%$$

False positive rate (FPR) measures the percentage of normal records incorrectly classified as attacks. It is calculated in Eq. (2):

(2) $$False\;Positive\;Rate\;\left(FPR\right)=\frac{FP}{TN\;+\;FP}*100\%$$

False negative rate (FNR) measures the percentage of attack records incorrectly classified as normal. It is calculated in Eq. (3):

(3) $$False\;Positive\;Rate\;\left(FNR\right)=\frac{FN}{FN+TP}*100\%$$

In the above equations, TP represents true positive, TN represents true negative, and FP refers to false positive, and FN refers to false negative.

In this study, six common classifiers were used: Logistic Regression (LR), SVM, Decision Tree (DT), Random Forest (RF), Naive Bayes (NB), and KNN. the reason for this set of classifiers because they learn in different ways (linear, non-linear patterns, probabilities, and distance), so the results are not dependent on a single type of model.

Tables 14 and 16 present the detection accuracy results for the resource usage dataset and the app permissions, sensors, and services dataset, respectively, using both testing approaches. Tables 15 and 17 present the false positive and false negative rates for the same classifiers and datasets.

**Table 14:** Detection accuracy of applying the classifiers to the resource usage datasets with the two different testing.

Dataset	Classifier	Test 1 (Cross-validation)		Test 2 (Supplied test set)	
		Cross-validation	Final Test	Internal testing	Unseen Test
Initial Compromise	LR	0.9852	0.9898	0.9868	0.9874
	SVM	0.9934	0.9957	0.9951	0.9945
	DT	0.9919	0.9937	0.9931	0.9918
	RF	0.9928	0.9945	0.9961	0.9925
	NB	0.9930	0.9949	0.9956	0.9933
	KNN	0.9923	0.9969	0.9936	0.9941

Credential Access	LR	0.9911	0.9945	0.9927	0.9922
	SVM	0.9960	0.9969	0.9966	0.9957
	DT	0.9917	0.9937	0.9922	0.9933
	RF	0.9929	0.9941	0.9936	0.9945
	NB	0.9930	0.9941	0.9946	0.9922
	KNN	0.9957	0.9965	0.9966	0.9953

Exfiltration	LR	0.9954	0.9965	0.9976	0.9961
	SVM	0.9960	0.9969	0.9971	0.9965
	DT	0.9922	0.9934	0.9932	0.9907
	RF	0.9930	0.9945	0.9937	0.9930
	NB	0.9930	0.9945	0.9951	0.9938
	KNN	0.9960	0.9969	0.9966	0.9965

**Table 15:** False positive and false negative rates of applying different classifiers to the resource usage datasets with the two different testing.

Dataset	Classifier	Test 1 (Cross-validation)				Test 2(Supplied test set)			
		Cross-validation		Final Test		Cross-validation		Final Test	
		FPR	FNR	FPR	FNR	FPR	FNR	FPR	FNR
Initial Compromise	LR	0.0221	0.0068	0.0153	0.0048	0.0227	0.0039	0.0173	0.0074
	SVM	0.0050	0.0083	0.0031	0.0056	0.0059	0.0039	0.0030	0.0082
	DT	0.0078	0.0085	0.0069	0.0056	0.0099	0.0039	0.0075	0.0090
	RF	0.0058	0.0087	0.0054	0.0056	0.0039	0.0039	0.0060	0.0090
	NB	0.0052	0.0089	0.0046	0.0056	0.0020	0.0068	0.0030	0.0106
	KNN	0.0071	0.0085	0.0008	0.0056	0.0089	0.0039	0.0038	0.0082

Credential Access	LR	0.0096	0.0080	0.0054	0.0056	0.0094	0.0051	0.0070	0.0087
	SVM	0.0000	0.0084	0.0000	0.0064	0.0000	0.0071	0.0000	0.0087
	DT	0.0082	0.0084	0.0061	0.0064	0.0094	0.0061	0.0047	0.0087
	RF	0.0059	0.0084	0.0054	0.0064	0.0057	0.0071	0.0023	0.0087
	NB	0.0009	0.0136	0.0008	0.0112	0.0009	0.0102	0.0000	0.0157
	KNN	0.0006	0.0084	0.0008	0.0064	0.0000	0.0071	0.0008	0.0087

Exfiltration	LR	0.0013	0.0081	0.0008	0.0063	0.0000	0.0052	0.0008	0.0070
	SVM	0.0000	0.0083	0.0000	0.0063	0.0000	0.0062	0.0000	0.0070
	DT	0.0075	0.0081	0.0069	0.0063	0.0073	0.0062	0.0116	0.0070
	RF	0.0059	0.0083	0.0046	0.0063	0.0064	0.0062	0.0070	0.0070
	NB	0.0000	0.0145	0.0000	0.0111	0.0000	0.0104	0.0000	0.0125
	KNN	0.0000	0.0083	0.0000	0.0063	0.0009	0.0062	0.0000	0.0070

**Table 16:** Detection accuracy of applying the classifiers to the apps-based datasets with the two different testing.

Dataset	Classifier	Test 1 (Cross-validation)		Test 2 (Supplied test set)	
		Cross-validation	Final Test	Internal testing	Unseen Test
Initial Compromise	LR	0.9764	0.9780	0.9819	0.9788
	SVM	0.9762	0.9737	0.9828	0.9737
	DT	0.9771	0.9780	0.9799	0.9796
	RF	0.9769	0.9761	0.9809	0.9757
	NB	0.9754	0.9757	0.9823	0.9757
	KNN	0.9736	0.9741	0.9789	0.9749

Privilege Escalation	LR	0.9758	0.9738	0.9721	0.9710
	SVM	0.9764	0.9734	0.9750	0.9726
	DT	0.9770	0.9734	0.9740	0.9749
	RF	0.9780	0.9765	0.9731	0.9730
	NB	0.9758	0.9702	0.9760	0.9702
	KNN	0.9766	0.9714	0.9755	0.9730

Exfiltration	LR	0.9751	0.9758	0.9786	0.9774
	SVM	0.9749	0.9762	0.9791	0.9782
	DT	0.9794	0.9770	0.9766	0.9747
	RF	0.9761	0.9739	0.9820	0.9758
	NB	0.9741	0.9762	0.9752	0.9758
	KNN	0.9760	0.9731	0.9766	0.9727

**Table 17:** False positive and false negative rates of applying different classifiers to the apps-based datasets with the two different testing.

Dataset	Classifier	Test 1 (Cross-validation)				Test 2(Supplied test set)			
		Cross-validation		Final Test		Internal testing		Unseen test	
		FPR	FNR	FPR	FNR	FPR	FNR	FPR	FNR
Initial Compromise	LR	0.0208	0.0266	0.0260	0.0177	0.0191	0.0171	0.0252	0.0169
	SVM	0.0249	0.0225	0.0337	0.0185	0.0201	0.0141	0.0344	0.0177
	DT	0.0229	0.0229	0.0245	0.0193	0.0201	0.0201	0.0230	0.0177
	RF	0.0236	0.0225	0.0298	0.0177	0.0211	0.0171	0.0321	0.0161
	NB	0.0009	0.0503	0.0046	0.0451	0.0000	0.0362	0.0046	0.0451
	KNN	0.0206	0.0326	0.0237	0.0282	0.0201	0.0221	0.0291	0.0209

Privilege Escalation	LR	0.0230	0.0255	0.0237	0.0289	0.0325	0.0231	0.0283	0.0297
	SVM	0.0239	0.0233	0.0245	0.0289	0.0335	0.0160	0.0306	0.0241
	DT	0.0235	0.0225	0.0230	0.0305	0.0316	0.0201	0.0283	0.0217
	RF	0.0215	0.0225	0.0214	0.0257	0.0354	0.0181	0.0298	0.0241
	NB	0.0013	0.0490	0.0008	0.0602	0.0019	0.0471	0.0008	0.0602
	KNN	0.0161	0.0313	0.0207	0.0369	0.0306	0.0181	0.0275	0.0265

Exfiltration	LR	0.0231	0.0268	0.0283	0.0198	0.0163	0.0268	0.0252	0.0198
	SVM	0.0277	0.0224	0.0329	0.0143	0.0211	0.0208	0.0260	0.0175
	DT	0.0205	0.0206	0.0237	0.0222	0.0191	0.0278	0.0252	0.0254
	RF	0.0244	0.0234	0.0314	0.0206	0.0172	0.0188	0.0237	0.0246
	NB	0.0057	0.0478	0.0054	0.0429	0.0000	0.0505	0.0031	0.0460
	KNN	0.0168	0.0317	0.0252	0.0286	0.0211	0.0258	0.0298	0.0246

As shown in Table 14, Table 15, Table 16, Table 17, the proposed DEFEAT datasets and features achieved high performance across all classifiers. For example, the resource usage dataset achieved detection accuracies of up to 99.60%, with FPR as low as 0.0227% and FNR as low as 0.0157%. The app permissions, sensors, and services dataset achieved detection accuracies of up to 98.28 %, with FPR as low as 0.0211 % and FNR as low as 0.0198%.

## Limitations

Although the DEFEAT datasets show promising results, some limitations should be reported. First, the datasets were collected from a physical Android device running Android Marshmallow (version 6). This version was selected to enable comprehensive, non-rooted access to device-level and app-based behavioral indicators, which are increasingly restricted in newer Android releases. These features are governed by the Linux kernel and the application execution model, which have remained stable across subsequent Android releases. Accordingly, the use of Android Marshmallow (version 6) represents a limitation in terms of cross-version generalizability. Second, although resource-usage features might be consistent in capturing behavioral deviations, the app-based dataset does not capture security mechanisms introduced in newer Android versions. These security mechanisms include custom or fine-grained runtime permission enforcement, background execution limits, and energy management mechanisms such as Doze mode. These OS-level controls can influence how applications request permissions, schedule background activities, and interact with system resources. Third, the malware samples used in this study are not intended to represent specific threat actors or nation-state campaigns. Instead, they serve as behavioral proxies for modelling common APT stage characteristics on Android devices. Although some samples belong to generic malware families, they exhibit behaviors that overlap with documented APT tactics, such as persistent background communication, access to sensitive resources, and external data transfer. Fourth, each application was executed for approximately 10 minutes. This duration is sufficient to capture the active behavioral manifestations of the simulated attack stages, during which malicious actions produce observable deviations in device behavior. However, it does not model the dormancy or long-term dwell periods characteristic of real-world APT campaigns. Finally, the simulation focuses on three stages of the APT lifecycle and is based on 36 malicious Android applications. While these stages are commonly observed in real-world attacks and the selected samples reflect multi-stage behavior aligned with the MITRE framework, they may not capture the full diversity of attack paths or malware variants encountered in operational environments.

## Ethics Statement

The authors have read and follow the ethical requirements for publication in Data in Brief. This work does not involve human subjects, animal experiments, or data collected from social media platforms.

## CRediT Author Statement

T.J. was responsible for the conceptualization, T.J. and A.A.A. were responsible design of the idea, methodology, analysis, validation, figure preparation, and writing of the original draft. M.M.S. contributed to validation, review, editing, resourcing, and funding acquisition. All authors reviewed and approved the final manuscript.

**Data Availability**

The DEFEAT datasets generated and analyzed during the current study are publicly available online at the following link: https://doi.org/10.17632/bdtn9vj7d7.3 (see Reference [1]).

## Figures and Tables

**Table A1:** Full Permissions Features with their Description.

Features	Description
Normal Permissions	
broadcast_sticky	Grants an app the ability to broadcast sticky intents
change_network_state	Grants an app the ability to alter network connectivity state
change_wifi_multicast_state	Grants an app the ability to enter Wi-Fi Multicast mode
change_wifi_state	Grants an app with the ability to change Wi-Fi connectivity state
disable_keyguard	Grants an app the ability to disable the lock screen if no secure authentication method is set
expand_status_bar	Grants an app the ability to expand/collapse the status bar
foreground_service	Grants an app the ability to utilize Service.startForeground
get_package_size	Grants an app the ability to retrieve information about the storage space utilized by any installed package on the device
install_shortcut	Grants an app the ability to install a shortcut in Launcher
internet	Grants an app the ability to open network sockets
kill_background_processes	Grants an app the ability to call ActivityManager.killBackgroundProcesses
manage_own_calls	Enables an application to implement its own calling interface and handle connection setup, audio routing, and call state management through the self-managed ConnectionService APIs
modify_audio_settings	Grants an app the ability to amend global audio settings
nfc	Grants an app the ability to execute I/O operations over NFC
read_sync_settings	Grants an app the ability to read the sync settings
read_sync_stats	Grants an app the ability to read the sync stats
receive_boot_completed	Grants an app the ability to receive the BOOT_COMPLETED broadcast intent, allowing it to perform actions automatically once the system has finished booting
reorder_tasks	Grants an app the ability to alter the Z-order of tasks
request_ignore_battery_optimizations	Grants an application the ability to request exemption from the system's battery optimization policies
set_wallpaper	Grants an app the ability to set the wallpaper
set_wallpaper_hints	Grants an app the ability to set the wallpaper hints
use_biometric	Grants an app the ability to use device supported biometric modalities
use_fingerprint	Grants an app the ability to use the device’s fingerprint sensor for authentication purposes
use_full_screen_intent	Grants an app the ability to display notifications using full-screen intents, typically used for high-priority events such as incoming calls or alarms that require immediate user attention
vibrate	Allows access to the vibrator
wake_lock	Grants an app the ability to utilize WakeLocks via the PowerManager API, preventing the processor from sleeping or the screen from dimming
write_sync_settings	Grants an app the ability to write the sync settings
Dangerous Permissions	
add_voicemail	Grants an app the ability to insert voicemails into the system
answer_phone_calls	Grants an app the ability to answer an incoming phone call
call_phone	Grants an app the ability to place phone calls directly, bypassing the Dialer interface and user confirmation
camera	Grants an app the ability to access the camera of the device
get_accounts	Grants app permission to access the list of user accounts registered on the device through the Accounts Service
read_calendar	Grants an app the ability to read the user's calendar data
read_call_log	Grants an app the ability to read the user's call log
read_contacts	Grants an app the ability to read the user's contacts data
read_external_storage	Grants an app the ability to read from external storage
read_logs	Grants an app the ability to read the low-level system log files
read_phone_numbers	Grants app permission to access the device phone numbers associated with the SIM card
read_phone_state	Grants an app read-only access to the device’s telephony state, including cellular network information, current call status, and registered phone accounts
read_sms	Grants an app the ability to read SMS messages
receive_mms	Grants an app the ability observes and process incoming MMS messages
receive_sms	Grants an app the ability to receive SMS messages
receive_wap_push	Grants an app the ability to receive WAP push messages
record_audio	Grants an app the ability to record audio.
send_respond_via_message	Grants app permission to delegate the 'respond via message' action for incoming calls to other applications
send_sms	Grants an app the ability to send SMS messages
write_calendar	Grants an app the ability to write the user's calendar data
write_call_log	Grants an app the ability to write and read the user's call log data
write_contacts	Allows the app to write data to the user's contact list, including adding new contacts or modifying existing entries
write_external_storage	Grants an app the ability to write to external storage
Signature Permissions	
install_packages	Grants an app the ability to install packages
manage_documents	Grants app permission to manage access to documents on the device
master_clear	Grants an app the ability to perform a factory reset of the device, erasing all user data, apps, and settings by invoking a master clear operation
modify_phone_state	Grants an app permission to modify telephony state, including actions such as powering on radio modules, issuing MMI (Man-Machine Interface) codes, and controlling other low-level telephony functions
mount_unmount_filesystems	Grants an app with the ability to mount and unmount file systems for removable storage
request_install_packages	Grants an application the ability to request the installation of application packages, typically by invoking the system package installer
set_animation_scale	Grants app permission to adjust the global animation scale settings
status_bar	Grants an app the ability to open, close, or disable the status bar and its icons
system_alert_window	Grants app permission to create overlay windows using TYPE_APPLICATION_OVERLAY, which appear on top of all other app interfaces
write_settings	Grants an app the ability to read or write the system settings
Privileged Permissions	
reboot	Grants app permission to initiate a device reboot
write_apn_settings	Grants app permission to modify app settings and access sensitive configuration fields, such as stored usernames and passwords of other applications
write_secure_settings	Grants an app the ability to read or write secure system settings

**Table A2:** Dictionary of technical keywords used in this study.

Keyword	Definition
Advanced Persistent Threat (APT)	A targeted cyberattack in which an adversary maintains long-term, covert access to a system or device to achieve objectives such as surveillance, credential theft, or data exfiltration.
Android runtime permissions	A permission model in which sensitive permissions are granted during app execution (runtime), based on user interaction, rather than only at install time.
App-based features	Features describing application-level behavior on Android, including permissions, sensors, and services accessed or requested during execution, as used in the DEFEAT app-based dataset.
Attack stage (multi-stage APT)	The attack path (e.g., Initial Compromise → Presence Expansion → Exfiltration) used to represent how attacker behavior evolves over time in a realistic sequence.
Command-and-Control (C&C)	Adversary communication is used to control a compromised device and coordinate actions such as issuing commands or receiving stolen data.
Confusion matrix (TP, TN, FP, FN)	A table summarizing classification outcomes: true positives, true negatives, false positives, and false negatives, used to compute performance metrics such as accuracy and error rates.
Cross-validation	A model evaluation approach that repeatedly splits data into training and testing folds to estimate performance stability across different partitions.
Credential Access stage	The stage in which the adversary is trying to steal account names, passwords, or other secrets that enable access to resources.
Dangerous permission	An Android permission protection level for operations that can expose sensitive user data or perform sensitive actions; typically requires explicit user approval (often at runtime).
Dataset “frame/instance/record”	One observation row in the dataset representing a snapshot of device or application behavior at a specific time interval (collected every 3 seconds in DEFEAT).
DEFEAT dataset	The proposed benchmarking dataset in this study consists of resource-usage features (CPU, RAM, battery, traffic) and app-based features (permissions, sensors, services), labelled for multi-stage APT analysis.
DEFENSE collector	The Android monitoring application developed in this study to record device-level and application-level behavioral features and transmit them to a server for storage and analysis.
Detection accuracy	The proportion of correctly classified instances across both classes (normal and attack), computed from the confusion matrix.
Exfiltration stage	The APT stage in which stolen data is transferred from the compromised device to external server, often observable through increased outbound communication.
False Negative (FN) / False Negative Rate (FNR)	FN: an attack instance predicted as normal. FNR is commonly computed as FN/(FN+TP) using confusion-matrix terms.
False Positive (FP) / False Positive Rate (FPR)	FP: a normal instance predicted as attack. FPR is commonly computed as FP/(FP+TN) using confusion-matrix terms.
Initial Compromise stage	The stage representing the attacker’s first foothold on the device (e.g., malicious app installed and activated), typically emphasizing stealth and minimal visible impact.
MITRE ATT&CK for Mobile	A structured knowledge base that organizes mobile adversary behavior into tactics/techniques, used as a threat-model reference to align simulated activities and labels.
Normalization	A preprocessing step that transforms features into comparable numeric ranges/scales so that no single feature dominates learning due to magnitude alone.
Permission protection level	Android’s categorization of permissions (e.g., normal/dangerous/signature/privileged) that reflects how restricted the capability is and how it can be granted.
Precision / Recall / F1-score	Standard metrics: recall is tp/(tp+fn) and F1 is the harmonic mean of precision and recall, widely used to evaluate the detection models.
Presence Expansion stage	A stage where adversary behavior broadens control and collects sensitive information (the paper operationalizes this via Privilege Escalation and/or Credential Access, depending on dataset component).
Privilege Escalation stage	Actions intended to gain higher privileges or broader control on the device (e.g., changing settings, weakening restrictions), represented as a stage label in the app-based dataset.
Resource-usage features	Device-level behavior indicators such as CPU usage, RAM usage, battery temperature/voltage, and network traffic (RX/TX/total), used in the DEFEAT resource-usage dataset.
Reverse TCP payload	A payload that initiates an outbound TCP connection from a compromised device to a command-and-control server, used in this study to establish controlled C&C communication during all simulated APT stages.
SHAP (SHapley Additive exPlanations)	An explainability method that attributes each feature’s contribution to a model prediction using Shapley-value principles, enabling interpretation of which features drive “attack vs normal.”
SHAP beeswarm plot	A SHAP visualization that shows the distribution of SHAP values per feature across instances, highlighting both impact direction and value density for the most influential features.
SMOTE	A class-balancing technique that synthetically generates new minority-class examples by interpolating between existing minority samples, commonly used to address class imbalance.
Supplied test set (unseen test)	A testing strategy that evaluates a trained model on a separate “unseen” subset that was not used for training, to estimate generalization in realistic settings.
Threat intelligence platform (VirusTotal)	A service that analyzes submitted files/URLs and aggregates multiple security engine results to support malware/indicator checking and validation.
t-test statistical analysis	A statistical test used to determine whether the mean values of a feature differ significantly between two groups (e.g., malware families), applied in this study to verify behavioral variability beyond payload effects.
TTP (Tactics, Techniques, and Procedures)	A common cyber threat-intelligence concept describing adversary stages (tactics), the methods used (techniques), and how they are executed in practice (procedures), often operationalized via ATT&CK-aligned analysis.

## Data Availability

Mendeley DataLabeled Multi-Stage Android APT Datasets (Original data).
